# Supplementary material for: Block-fitness modeling of the global air mobility network
Source: Nat Commun. 2026 Oct 12;17:11559. doi: 10.1038/s41467-026-78115-6 (PMC13748577; doi:10.1038/s41467-026-78115-6)
Supplement: Supplementary file 1 [file 41467_2026_78115_MOESM1_ESM.pdf]

Block-Fitness Modeling of the Global Air Mobility Network

Giulia Fischetti[1,2,+], **Anna Mancini**[3,4,+], **Giulio Cimini**[3,4,5,*], **Jessica T. Davis**[6], **Abby O. K. Leung**[6], **Alessandro Vespignani**[6], **and Guido Caldarelli**[5,1,7,8,*]

[1]DMSN, Ca' Foscari University of Venice, 30172 Mestre (Italy)
[2]IAMP, ZHAW School of Engineering, 8400 Winterthur (Switzerland)
[3]Physics Dept. and INFN, University of Rome Tor Vergata, 00133 Rome (Italy)
[4]Enrico Fermi Research Center, 00184 Rome (Italy)
[5]CNR-Institute for Complex Systems (ISC), 00185 Rome (Italy)
[6]MOBS Lab, Northeastern University, Boston MA 02115 (USA)
[7]ECLT Ca' Bottacin, 30123 Venice (Italy)
[8]LIMS, Royal Institution, London W1S4BS (United Kingdom)
[*]Corresponding authors: G. Cimini (giulio.cimini@roma2.infn.it), G. Caldarelli (guido.caldarelli@unive.it)
[+]These authors contributed equally to this work

Supplementary Information

Supplementary Methods

WAN data

The global air-travel data come from the Official Aviation Guide (OAG)[1], which reports the number of passengers flying between airport pairs each month. Specifically, data report the number of people traveling between airport pairs and count each trip only once, from the starting airport to the final destination (ignoring layovers). To generate the basin-level network we aggregated data from September to November 2019. We build a seasonal flight network by averaging the passenger flows from these three months. Airports are grouped into larger geographic "basins" which are generated from a Voronoi tessellation centered around major transportation hubs. From this network we can calculate the daily probability of travel between two basin pairs, which is used in the metapopulation model to simulate the global spread of a pathogen. This mobility network has been used previously to study the global spread of infectious diseases[2].

The Haversine formula is used to compute the great-circle distance d between two points on the surface of a sphere given their latitudes and longitudes. In the formula:

$$h = \sin^2\left(\frac{\phi_2 - \phi_1}{2}\right) + \cos(\phi_1)\cos(\phi_2)\sin^2\left(\frac{\lambda_2 - \lambda_1}{2}\right),$$
$$d = 2r \arcsin\left(\sqrt{h}\right),$$

where the variables are defined as follows:

- ϕ_1, ϕ_2: The latitudes of the first and second points, respectively, measured in radians.
- λ_1, λ_2: The longitudes of the first and second points, respectively, measured in radians.
- r: The radius of the sphere (for Earth, r is approximately 6,371 km).
- h: An auxiliary value that represents the square of half the chord length between the points. It is computed using the differences in latitudes and longitudes.
- d: The great-circle distance between the two points along the surface of the sphere.

Model formulations

Here we describe different model formulations we tested for reconstructing the WAN, for the general case of weighted directed networks. Each link $i \to j$ has weight $w_{i\to j}$ (in general $\neq w_{j\to i}$), hence each node i is characterized by a value of in-strength $s_i^{\text{in}} = \sum_j w_{j\to i}$ and out-strength $s_i^{\text{out}} = \sum_j w_{i\to j}$. If the network is undirected (meaning $w_{i\to j} = w_{j\to i} \equiv w_{ij}$), there is only one strength value per node, hence it is enough to replace $s_i^{\text{out}} = s_i^{\text{in}}$ with $s_i = \sum_j w_{ij}$ (for models **B** and **C**, this holds also for block-specific strengths).

Model K. The fitness-induced density-corrected Gravity Model, proposed in[3] to reconstruct economic and financial networks, is based on two inference steps. First, links are drawn according to the canonical *configuration model* (CM) prescription, relying on a fitness assumption that nodes with large passenger flux are likely to be connected. Hence the CM parameters controlling degrees are replaced with node strengths. Then each link $i \to j$ (with $i \neq j$) is sampled independently according to the connection probability

$$p_{i\to j}^{(\mathbf{K})} = \frac{z^{(\mathbf{K})} s_i^{\text{out}} s_j^{\text{in}}}{1 + z^{(\mathbf{K})} s_i^{\text{out}} s_j^{\text{in}}} \tag{1}$$

which depends on s_i^{out} and s_j^{in} (the required inputs of the model). The only free parameter, $z^{(\mathbf{K})}$, is determined by setting the expected number of links of the network to the empirical value: $L_{tot} = \sum_i \sum_{j(\neq i)} p_{i\to j}^{(\mathbf{K})}$ (see Supplementary Table 1). Second, every sampled link $i \to j$ (realized with probability $p_{i\to j}^{(\mathbf{K})}$) is assigned weight

$$w_{i\to j}^{(\mathbf{K})} = \frac{s_i^{\text{out}} s_j^{\text{in}}}{W \cdot p_{i\to j}^{(\mathbf{K})}} \tag{2}$$

where $W = \sum_i s_i^{\text{out}} = \sum_i s_i^{\text{in}}$ is the total weight of the network, and $w_{i\to j}^{(\mathbf{K})} = 0$ otherwise. The model thus generates sparse topologies but, at the same time, the average link weight reads $\langle w_{i\to j}^{(\mathbf{K})} \rangle = s_i^{\text{out}} s_j^{\text{in}} / W$, analogously to what prescribed by standard maximum-entropy gravity models[4,5]. Therefore the model preserves, as ensemble averages, the in- and out- strength of each node: $\langle s_i^{\text{out}} \rangle = \sum_j \langle w_{i\to j}^{(\mathbf{K})} \rangle \equiv s_i^{\text{out}}$ and $\langle s_i^{\text{in}} \rangle = \sum_j \langle w_{j\to i}^{(\mathbf{K})} \rangle \equiv s_i^{\text{in}}$.

Model S. For sparse networks, the fitness ansatz of model **K** actually means assuming that degrees are linearly proportional to strengths. However for the WAN it is known that $s(k) \sim k^\beta$ with $\beta > 1$[6–8]. We can thus employ a non-linear fitness ansatz of this form, leading to model formulation **S** where the connection probability takes the form:

$$p_{i\to j}^{(\mathbf{S})} = \frac{z^{(\mathbf{S})} [s_i^{\text{out}} s_j^{\text{in}}]^\alpha}{1 + z^{(\mathbf{S})} [s_i^{\text{out}} s_j^{\text{in}}]^\alpha}. \tag{3}$$

and the parameter $z^{(\mathbf{S})}$ is obtained as before (Supplementary Table 1). We set the optimal exponent α by minimizing the RMSD between the empirical and reconstructed degree sequences (Supplementary Figure 2). For weights we keep the same functional form of eq. (2): $w_{i\to j}^{(\mathbf{S})} = s_i^{\text{out}} s_j^{\text{in}} \Big/ \left(W \cdot p_{i\to j}^{(\mathbf{S})}\right)$ with probability $p_{i\to j}^{(\mathbf{S})}$ and $w_{i\to j}^{(\mathbf{S})} = 0$ otherwise, hence also model **S** preserves, as ensemble averages, the in- and out- strength of each node. In the regime of very sparse networks, where $p_{i\to j}^{(\mathbf{S})} \sim z^{(\mathbf{S})} [s_i^{\text{out}} s_j^{\text{in}}]^\alpha$, this leads to $w_{i\to j}^{(\mathbf{S})} \sim (k_i^{\text{out}} k_j^{\text{in}})^{\beta(1-\alpha)}$, with values of the exponent compatible with empirical findings of[6].

Model D. In order to introduce a dependency on the distance between airports, we follow the maximum entropy construction for spatial networks[9–11], prescribing to constrain the node degrees and the number of links at a certain distance interval. Merging this recipe with the fitness ansatz leads to model formulation **D**:

$$p_{i\to j}^{(\mathbf{D})} = \frac{z^{(\mathbf{D})} s_i^{\text{out}} s_j^{\text{in}} \, l(d_{ij})}{1 + z^{(\mathbf{D})} s_i^{\text{out}} s_j^{\text{in}} \, l(d_{ij})} \tag{4}$$

where d_{ij} is the distance between nodes i and j, $l(d)$ is the probability of having a link between nodes at distance d, and $z^{(\mathbf{D})}$ is obtained as before (Supplementary Table 1). Note that the function $l(d)$ is related to the cost of establishing a link between nodes at distance d; if $l(d)$ is exponential then the cost is linear in d, while a power-law $l(d)$ means that the cost is logarithmic in d[11]. Weights are again assigned as for eq. (2): $w_{i\to j}^{(\mathbf{D})} = s_i^{\text{out}} s_j^{\text{in}} \Big/ \left(W \cdot p_{i\to j}^{(\mathbf{D})}\right)$ with probability $p_{i\to j}^{(\mathbf{D})}$ and $w_{i\to j}^{(\mathbf{D})} = 0$ otherwise. Hence model **D** weights do depend on the distance through the connection probabilities, while still preserving on average the in- and out- strength of each node.

Model B (used in the main text). In order to impose a community structure on the model network while keeping the degree constraints, we employ a composition of models **K** (one for each block). We thus split the network nodes into a set of communities $\{g\}$; then we endow each node i with $|g|$ block-specific values of in- and out-strength, $s^{\text{in}}_{g\to i} = \sum_{j(\neq i)\in g} w_{j\to i}$ and $s^{\text{out}}_{i\to g} = \sum_{j(\neq i)\in g} w_{i\to j}$, quantifying the connectivity of i with respect to nodes in community g. The probability of connection from node i to node j, belonging respectively to community g_i and g_j, thus reads:

$$p^{(\mathbf{B})}_{i\to j} = \frac{z^{(\mathbf{B})}_{g_i g_j} s^{\text{out}}_{i\to g_j} s^{\text{in}}_{g_i\to j}}{1 + z^{(\mathbf{B})}_{g_i g_j} s^{\text{out}}_{i\to g_j} s^{\text{in}}_{g_i\to j}} \tag{5}$$

where the parameters $z^{(\mathbf{B})}_{g_i g_j}$ can be computed through the set of equations $\sum_{i\in g_i}\sum_{j\in g_j} p^{(\mathbf{B})}_{i\to j} = L_{g_i,g_j}$ (Supplementary Figure 1). Weights are then assigned as $w^{(\mathbf{B})}_{i\to j} = s^{\text{out}}_{i\to g_j} s^{\text{in}}_{g_i\to j} \Big/ \left(W_{g_i g_j}\cdot p^{(\mathbf{B})}_{i\to j}\right)$ with probability $p^{(\mathbf{B})}_{i\to j}$ and $w^{(\mathbf{B})}_{i\to j} = 0$ otherwise, where now $W_{g_i g_j} = \sum_{i\in g_i}\sum_{j\in g_j} w_{i\to j}$ is the total weight of connections from nodes in g_i to nodes in g_j. Therefore the model preserves, as ensemble averages, the out-strength and the in-strength by group of each node: $\langle s^{\text{in}}_{g_i\to j}\rangle = \sum_{i\in g_i}\langle w^{(\mathbf{B})}_{i\to j}\rangle \equiv s^{\text{in}}_{g_i\to j}$ and $\langle s^{\text{out}}_{i\to g_j}\rangle = \sum_{j\in g_j}\langle w^{(\mathbf{B})}_{i\to j}\rangle \equiv s^{\text{out}}_{i\to g_j}$.

Model C. The combined model that incorporates all ingredients of the previous formulations is based on the same community structure $\{g\}$ of model **B**, so that the probability of connection from node i to node j, belonging respectively to community g_i and g_j, is:

$$p^{(\mathbf{C})}_{i\to j} = \frac{z^{(\mathbf{C})}_{g_i g_j} [s^{\text{out}}_{i\to g_j} s^{\text{in}}_{g_i\to j}]^{\alpha} l(d_{ij})}{1 + z^{(\mathbf{C})}_{g_i g_j} [s^{\text{out}}_{i\to g_j} s^{\text{in}}_{g_i\to j}]^{\alpha} l(d_{ij})} \tag{6}$$

where $l(d_{ij})$ is the geographic distance between nodes i and j, while the optimal exponent α is set to minimize the RMSD between the empirical and reconstructed degree sequences (Supplementary Figure 2). The parameters $z^{(\mathbf{C})}_{g_i g_j}$ can be computed through the set of equations $\sum_{i\in g_i}\sum_{j\in g_j} p^{(\mathbf{C})}_{i\to j} = L_{g_i,g_j}$ (Supplementary Figure 1). Weights are then assigned as $w^{(\mathbf{C})}_{i\to j} = s^{\text{out}}_{i\to g_j} s^{\text{in}}_{g_i\to j} \Big/ \left(W_{g_i g_j}\cdot p^{(\mathbf{C})}_{i\to j}\right)$ with probability $p^{(\mathbf{C})}_{i\to j}$ and $w^{(\mathbf{C})}_{i\to j} = 0$ otherwise. Thus model **C** preserves, as ensemble averages, the out-strength and the in-strength by group of each node, as model **B** does.

Model R. As benchmark we consider model **R** of a random network with the same density of WAN, hence $p^{(\mathbf{R})}_{i\to j} \equiv \bar{p} = L_{tot}/[N(N-1)]\ \forall i,j$. This results in $\langle k^{(\mathbf{R})}_i\rangle = L_{tot}/N\ \forall i$. For weights, $w^{(\mathbf{R})}_{i\to j} = s^{\text{out}}_i s^{\text{in}}_j/(W\bar{p})$ with probability $\bar{p}$, hence also model **R** preserves node strengths as ensemble averages.

Model	Equation	z (basin)	residual error	z (airport)	residual error
**K**	$\sum_{ij} a_{i\to j} = \sum_i \sum_{j(\neq i)} \frac{z^{(\mathbf{K})} s_i^{\text{out}} s_j^{\text{in}}}{1+z^{(\mathbf{K})} s_i^{\text{out}} s_j^{\text{in}}}$	$5.70 \cdot 10^{-9}$	$-1 \cdot 10^{-12}$	$2.44 \cdot 10^{-11}$	$-5 \cdot 10^{-11}$
**S**	$\sum_{ij} a_{i\to j} = \sum_i \sum_{j(\neq i)} \frac{z^{(\mathbf{S})} [s_i^{\text{out}} s_j^{\text{in}}]^{\alpha}}{1+z^{(\mathbf{S})} [s_i^{\text{out}} s_j^{\text{in}}]^{\alpha}}$	$1.69 \cdot 10^{-7}$	$-1 \cdot 10^{-11}$	$1.33 \cdot 10^{-8}$	$2 \cdot 10^{-11}$
**D**	$\sum_{ij} a_{i\to j} = \sum_i \sum_{j(\neq i)} \frac{z^{(\mathbf{D})} s_i^{\text{out}} s_j^{\text{in}} l(d_{ij})}{1+z^{(\mathbf{D})} s_i^{\text{out}} s_j^{\text{in}} l(d_{ij})}$	$8.58 \cdot 10^{-7}$	$5 \cdot 10^{-12}$	$1.57 \cdot 10^{-9}$	$2 \cdot 10^{-11}$

Supplementary Table 1. For models **K**, **S** and **D**, the parameter z is determined by equating the total number of links of the model network, namely $\sum_i \sum_{j(\neq i)} p_{i\to j}$, with the number of links of the real network $L_{tot} = \sum_i \sum_{j(\neq i)} a_{i\to j}$. Here we report the specific equation for each model and the corresponding value of z.

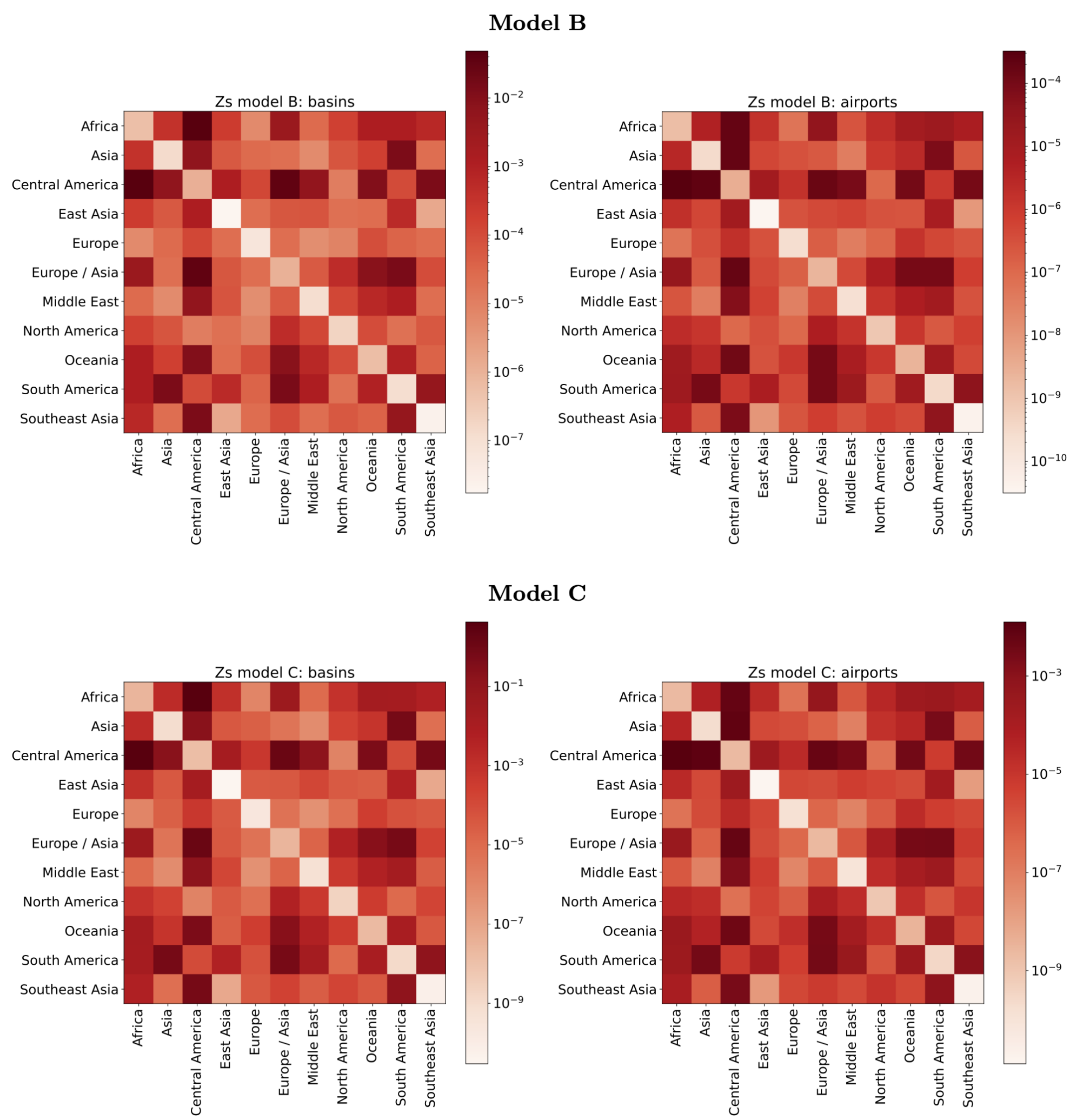

Supplementary Figure 1. For models **B** and **C**, the parameter $z_{g_i g_j}$ for each pair of communities $(g_i g_j)$ is determined by equating the total number of links of the model network, namely $\sum_{i\in g_i} \sum_{j(\neq i)\in g_j} p_{i\to j}$, with the number of links of the real network $L_{g_i g_j} = i \in g_i \sum_{j(\neq i)\in g_j} a_{i\to j}$. Here we report heat maps with the corresponding values of z for each pair of regions.

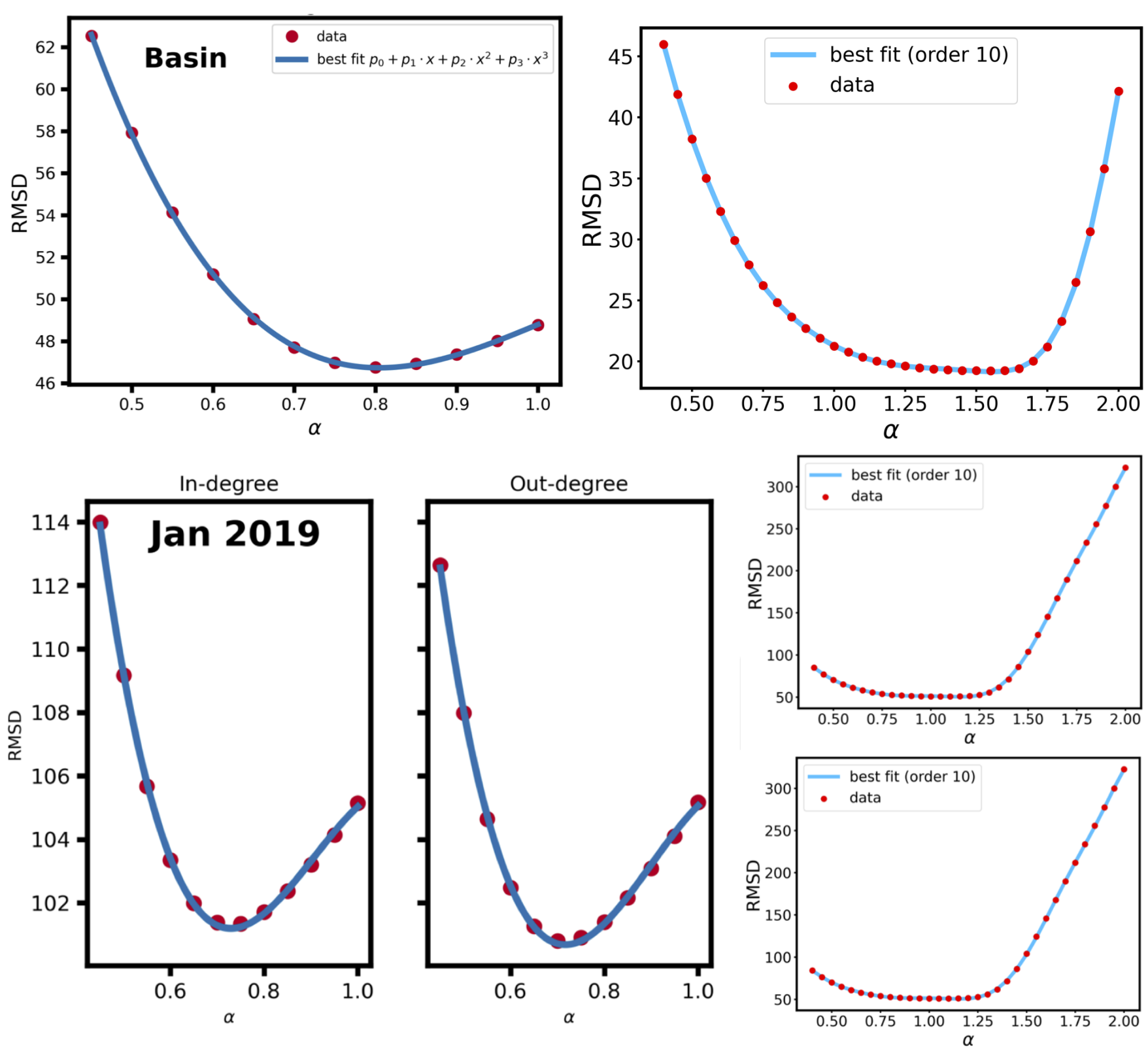

Supplementary Figure 2. We obtain the optimal α exponent for models **S** and **C** by minimizing the RMSD between the real and reconstructed degree sequence as a function of α. Basins dataset (upper plots): for **S** (left plot) we fitted the curve with a 3rd degree polynomial, yielding the minimum value $\alpha = 0.80$; for **C** (right plot) we used a 10th degree polynomial, yielding $\alpha = 1.56$. Airport dataset (lower plots): for **S** (left plot), $\alpha = 0.72$; for **C** (right plot), $\alpha = 1.16$.

Supplementary Note 1

Results for the different model formulations (basins dataset)

Here we discuss and compare the results of all model formulations in reconstructing the topological and dynamical features of the WAN (refer to Supplementary Tables 2 and 3 for an overview of the results and the following plots for more detailed comparison). Firstly, by construction all models can reproduce the strength sequence. Concerning the reconstruction of degree values (a baseline requirement, supporting the validity of the ansatz), all models perform fairly well, except of course model **R** that generates a network with constant average degree and achieves a very low recall of 0.018 (i.e., the value of the network density).

With respect to the benchmark model **R**, the simple model **K** brings a very large improvement on recall as well as on the agreement between model and empirical link probabilities. Furthermore, it can well interpolate the degree vs strength relation but is not able to capture its dispersion. However, it fails in reproducing both the dependence of link occurrence on distance and the community structure of the network. Concerning epidemic simulations, **K** generally improves with respect to the random benchmark **R**, but tends to anticipate the epidemic peak and leads to a more homogeneously diffused prevalence map than simulations on the real network.

Model **S**, incorporating the super-linear relation between strengths and degrees, improves on **K** in reconstructing degrees and connection probabilities, but leads to worse recall and reconstruction of topological features: it slightly improves in the interpolation of the relation k vs s but is not able to capture its dispersion; moreover it fails in reproducing both the dependence on distance and the community structure of the network. Model **D**, designed to capture the dependence of connection probabilities on geographical distances, shows a rather opposite behavior. With respect to **K**, the reconstruction of connection probabilities and degrees does not improve while recall increases substantially. Notably, **D** also captures some of the dispersion of the k vs s relation, but not the community structure of the network and, importantly, is not able to improve in reproducing the dependence of link probability on distance. This suggests that air travel (as described by origin-destination flows) is not much affected by the underlying metric space, but rather depends on the geographic area of origin and destination (constrained by model **B**). Concerning epidemic simulations, both models **S** and **D** slightly improve on model **K**, but outcomes remain rather distinct from those simulated on the empirical network.

As discussed in the main text, model **B** improves in any aspect with respect to all other models. Interestingly, the full model **C** (incorporating, at the same time, the ingredients of **S**, **D**, **B**) does not perform as well as model **B**. This suggests that the presence of multiple determinants for links presence is adding noise rather than improving the signal.

Models	RMSRE_s	RMSRE_k	RMSRE_p	RMSRE_w	Recall	β	$\mathrm{MSE}_{l(d)}$	M
K	10^{-3}	0.34	0.030	0.17	0.31	1.10(5)	9.52	0.0
S	10^{-3}	0.18	0.004	0.17	0.26	1.35(4)	17.5	0.0
D	10^{-3}	0.32	0.020	0.17	0.40	1.07(3)	7.97	0.0
B	10^{-2}	0.18	0.016	0.063	0.75	1.17(0)	1.61	0.63
C	10^{-2}	0.26	0.022	0.089	0.64	0.91(2)	0.60	0.63
R	10^{-3}	10	0.98	0.073	0.018	(4)	157	0.0

Supplementary Table 2. Reconstruction of network properties. (Left sector) Values of Root Mean Squared Relative Error $\sqrt{n^{-1}\sum_i(\langle y_i\rangle/y_i-1)^2}$ of reconstructed quantities $\langle y_i\rangle$ against empirical observations y_i, where y values are averages over histogram bins. RMSRE_s of model strengths $\langle s_i\rangle=\sum_{j\neq i}\langle w\rangle_{ij}$ versus empirical strengths $s_i=\sum_{j\neq i}w_{ij}$; RMSRE_k of model degrees $\langle k_i\rangle=\sum_{j\neq i}p_{ij}$ versus empirical degrees $k_i=\sum_{j\neq i}a_{ij}$; RMSRE_p of empirical link probability f_{ij} versus model link probability p_{ij}; RMSRE_w of average model weights $\langle w\rangle_{ij}$ versus empirical weights w_{ij}. Recall values for link prediction, obtained using the model link probability to determine the outcome of a binary classification test on the existence of a link. (Left sector) OLS fit exponent β of the strengths vs degrees relation (uncertainties are given in parentheses and refer to the last significant digit); for real data, $\beta=1.2(2)$. Mean Squared Error (MSE) between empirical and model curve of link probability as a function of distance. Average modularity M of the network; for real data, $M=0.64$.

Model	Δ area	L2[i]	Peak time	Peak magn.	Epid. length	RMSE[i_m]	L2[H]
K	2.2 ± 0.8	0.29 ± 0.10	9.2 ± 4.4	0.016 ± 0.009	14.6 ± 4.6	0.060 ± 0.007	20.2 ± 4.9
S	2.0 ± 0.8	0.27 ± 0.11	8.3 ± 4.2	0.016 ± 0.008	11.0 ± 5.0	0.058 ± 0.008	17.3 ± 5.1
D	2.0 ± 0.8	0.27 ± 0.10	8.1 ± 4.0	0.015 ± 0.010	9.5 ± 4.7	0.064 ± 0.009	16.6 ± 5.4
B	0.56 ± 0.31	0.073 ± 0.040	2.3 ± 1.8	0.0048 ± 0.0035	4.5 ± 3.2	0.050 ± 0.006	14.2 ± 6.2
C	0.61 ± 0.34	0.080 ± 0.043	2.3 ± 1.8	0.0050 ± 0.0038	7.0 ± 3.0	0.063 ± 0.010	17.6 ± 6.9
R	2.4 ± 1.1	0.31 ± 0.13	8.6 ± 5.2	0.012 ± 0.008	8.5 ± 6.3	0.079 ± 0.019	21.8 ± 8.4
Data	0.43 ± 0.32	0.058 ± 0.042	2.0 ± 1.7	0.0043 ± 0.0034	2.2 ± 2.2	0.024 ± 0.005	13.1 ± 6.3

Supplementary Table 3. Similarity of epidemic simulations on real and model networks. Given a pair of simulations, respectively on the *real* network and a *model* instance, we compute: the area between the prevalence curves, $\Delta=\sum_t|i(t)_{real}-i(t)_{model}|$; their L2 distance $\mathrm{L2}[i]=\sqrt{\sum_t[i(t)_{real}-i(t)_{model}]^2}$; the peak time difference $\Delta t^*=|t^*_{real}-t^*_{model}|$, where $t^*_{real}=\arg\max_t i(t)_{real}$ and similarly for t^*_{model}; the peak magnitude difference $\Delta i^*=|i^*_{real}-i^*_{model}|$, with $i^*_{real}=\max_t i(t)_{real}$ and similarly for i^*_{model}; the difference in epidemic lengths $\Delta L=|L_{real}-L_{model}|$, where $L_{real}=(t^{\mathrm{end}})_{real}-(t^{\mathrm{start}})_{real}$ and $(t^{\mathrm{start}})_{real}$ and $(t^{\mathrm{end}}_{real})$ denote the first and last times at which $i(t)_{real}$ exceeds a small threshold ε (and analogously for L_{model}); RMSE of prevalence vector averaged over time, $\mathrm{RMSE}[i_m]=T^{-1}\sum_t\sqrt{N^{-1}\sum_m[(i_m(t))_{real}-(i_m(t))_{model}]^2}$; normalized L2 distance of the entropy curves, $\mathrm{L2}[H]=\sqrt{\sum_t[H(t)_{real}-H(t)_{model}]^2}$. Values in each row are averages over pairwise comparisons of a simulation on the empirical network (x5) and on a network generated by the corresponding model (x20), for each of the 24 seed basins. "Data" values instead are averages over pairwise comparisons of simulations on the empirical network (x5), for each of the 24 seed basins. Uncertainties are given as standard deviations of pairwise comparisons.

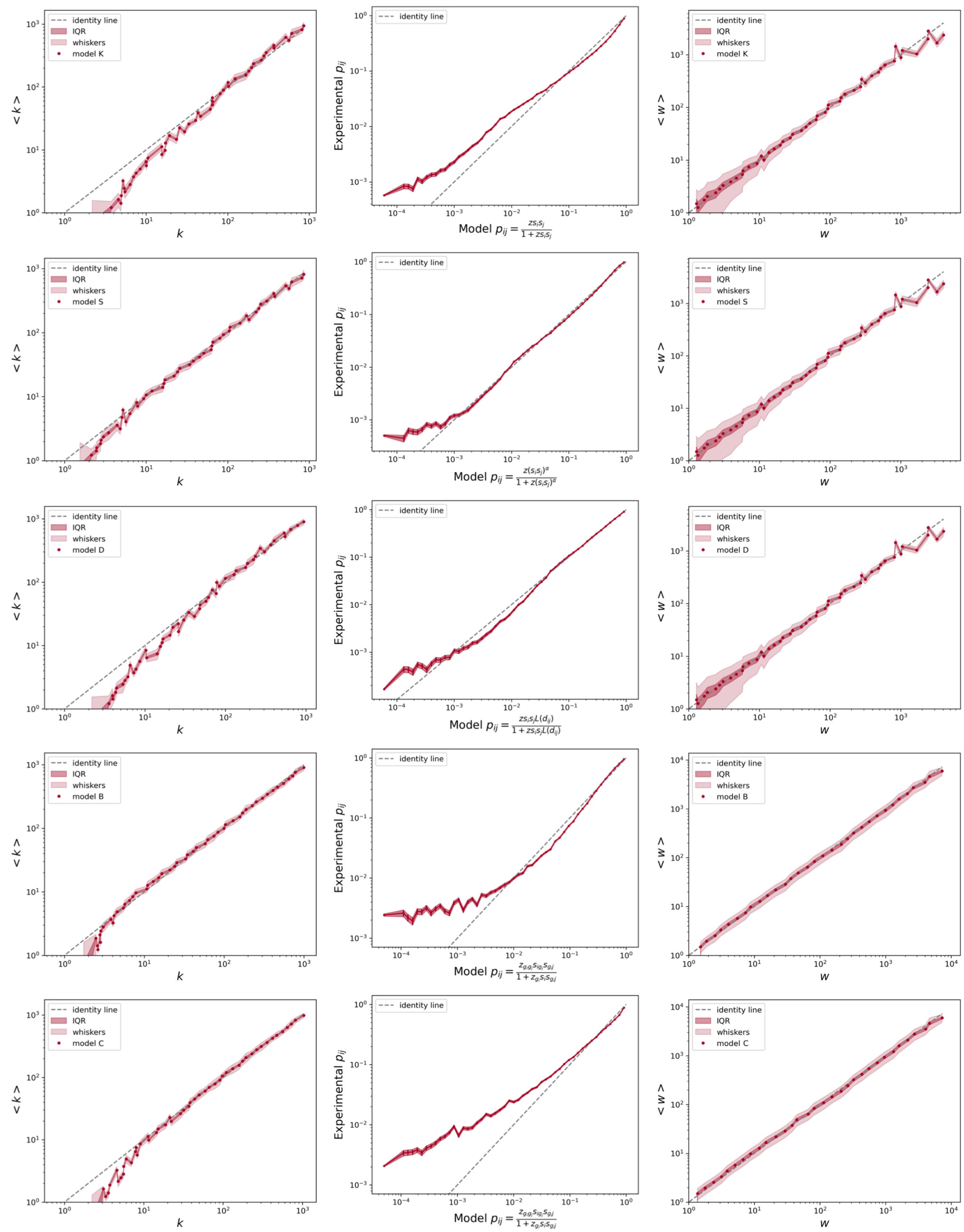

Supplementary Figure 3. Reconstruction of binary and weighted properties of the WAN. From top to bottom: Models **K**, **S**, **D**, **B**, **C** (Model **R** is not shown). The dashed identity line marks the perfect agreement between empirical and model values. The shaded regions represent the interquartile range and the whiskers of the set of model values. Left column: Model degrees versus observed degrees. Central column: Observed link probability as a function of model link probability. Right column: Model weights versus observed weights.

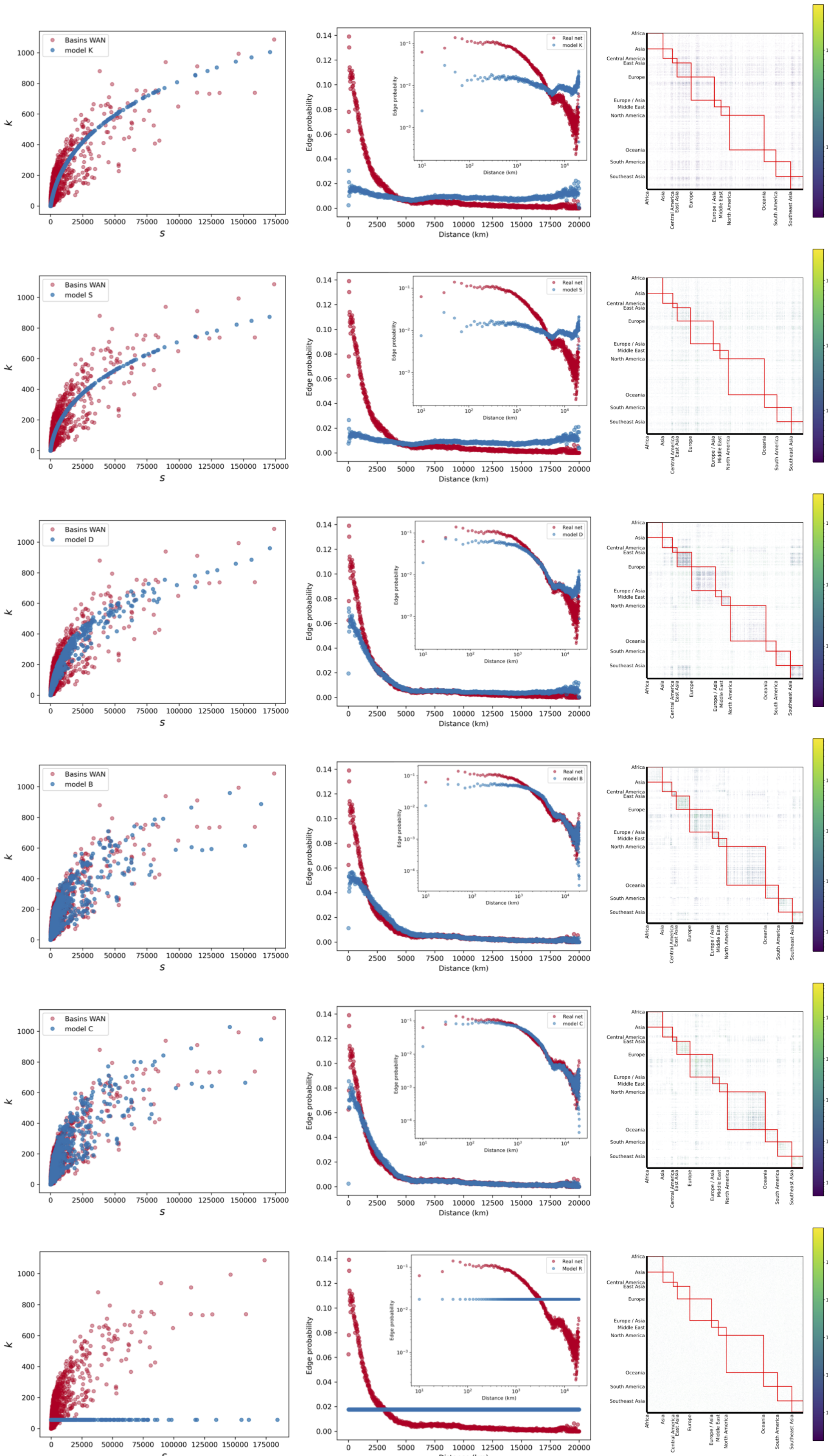

Supplementary Figure 4. Properties of the reconstructed networks. From top to bottom: Models **K**, **S**, **D**, **B**, **C**, **R**. Left column: degrees vs strengths. Central column: link probability as a function of distance. Right column: community structure.

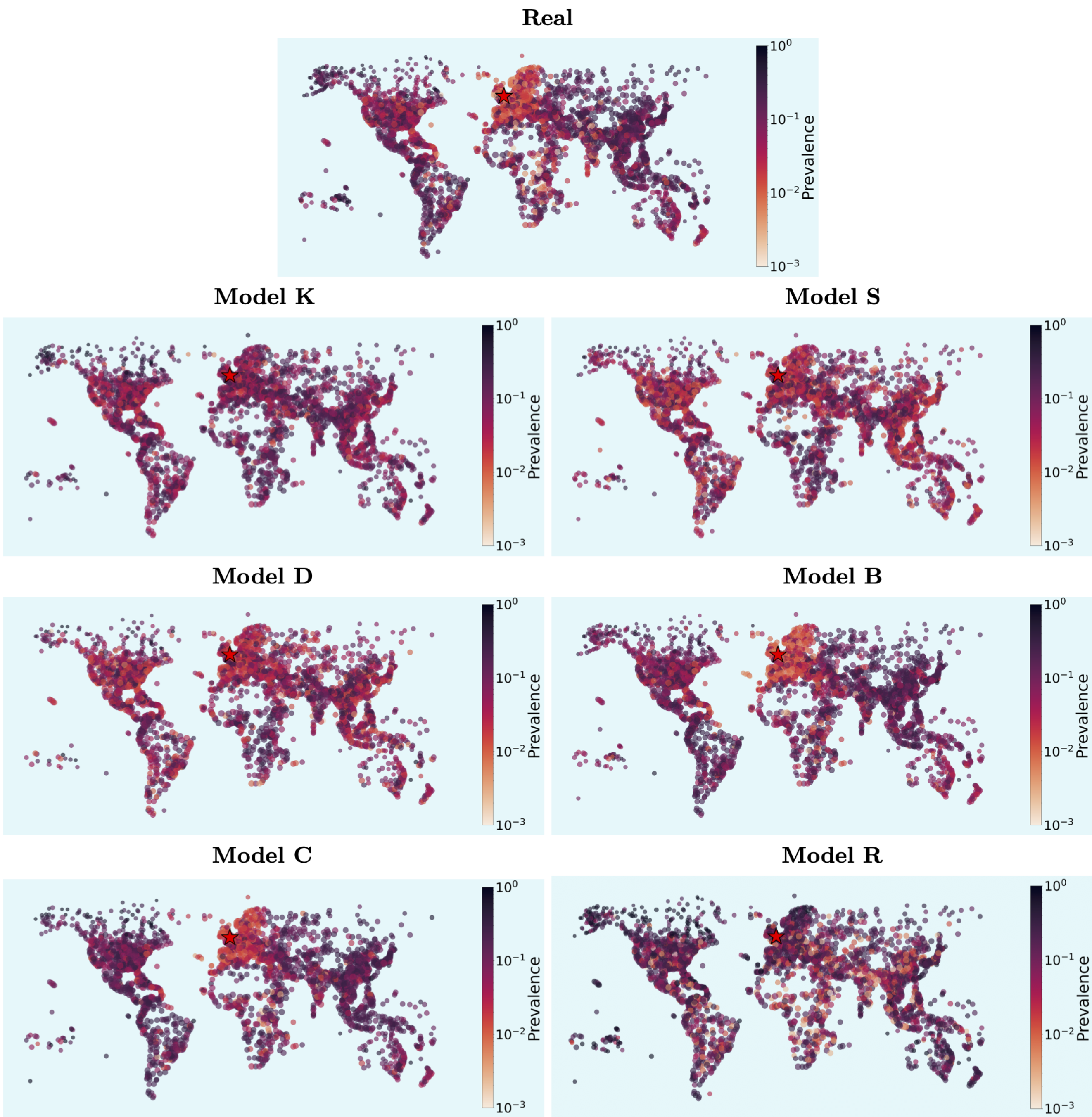

Supplementary Figure 5. Prevalence maps for the real and model WAN, concerning simulations with seed=London, at time step $t = 80$.

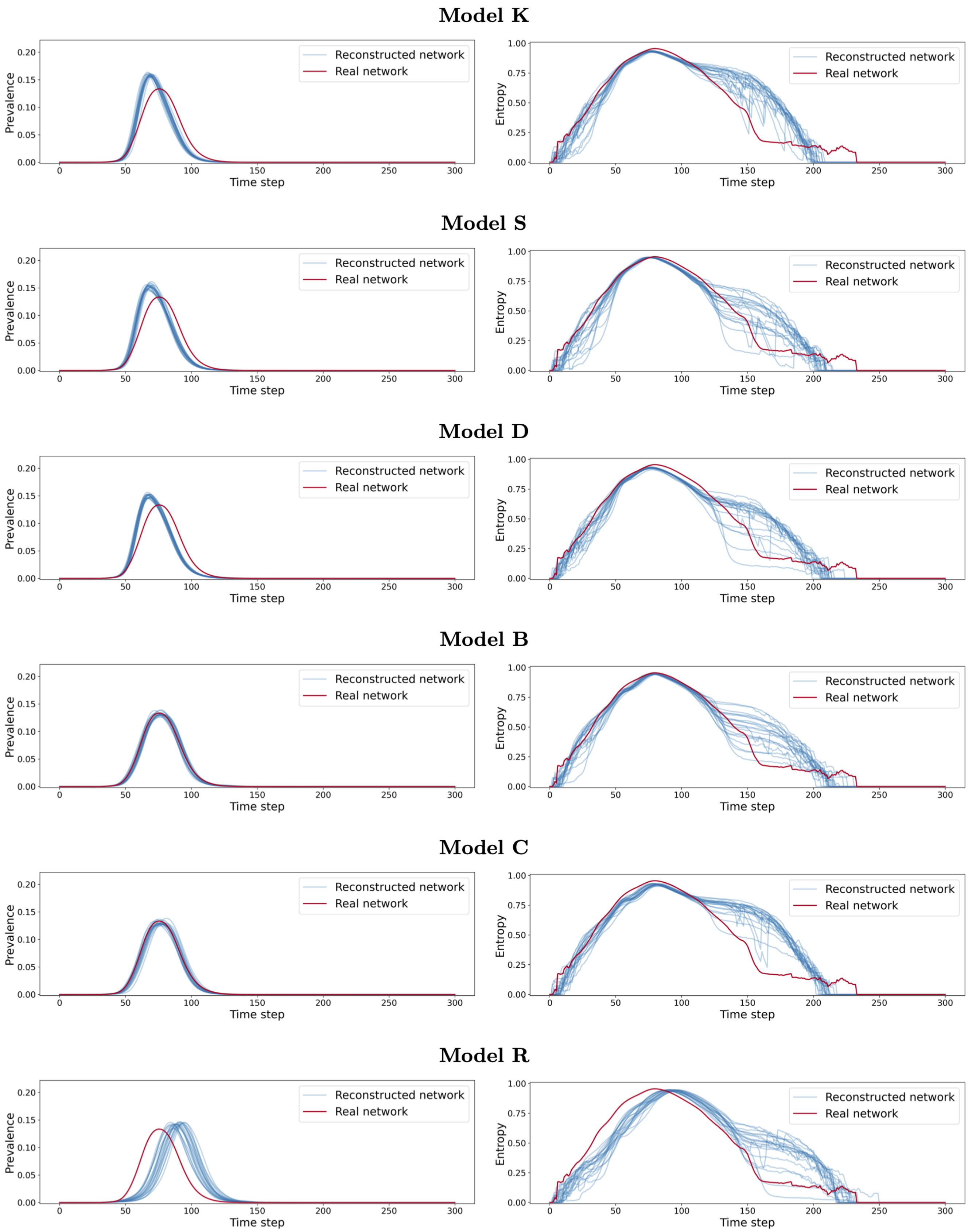

Supplementary Figure 6. Metapopulation results for seed = London. Comparison of one real simulation and 20 model simulations, in terms of the time evolution of total prevalence and entropy of the prevalence distribution.

Supplementary Note 2

Results for the airport-based WAN

We also consider the WAN representation where nodes correspond to individual airports and links denote the flow of passengers between airports. The network is directed in this case, meaning that the weight of the link $w_{i\to j}$ is the number of passengers traveling from airport i to airport j per month (again ignoring layovers). Here we consider passengers traveling in January of 2019 and airports that send out at least 100 travelers per month. The in- and out-strength of node i, given by $s_i^{\text{in}} = \sum_j w_{j\to i}$ and $s_i^{\text{out}} = \sum_j w_{i\to j}$, represent the number of incoming and outgoing passengers at the corresponding airport, whereas, the in degree $k_i^{\text{in}} = \sum_j a_{j\to i}$ and out degree $k_i^{\text{out}} = \sum_j a_{i\to j}$ are the number of direct connections from and to other airports ($a_{i\to j} = 1$ if $w_{i\to j} > 0$ and zero otherwise). Note that most of the links are reciprocal[12] (the link reciprocity coefficient is 0.9999 and the correlation between the weights of opposed links is 0.9978). The geographic distance between airports i and j is obtained using the Haversine formula, while the geographic communities are defined as for the basin dataset. Results of the reconstruction exercise for such data (reported below) confirm the findings obtained for the basin representation of the WAN, concerning the topological reconstruction of the network. Metapopulation simulations cannot be performed with this representation, as we cannot associate a population value to an individual airport.

Model	$\text{RMSRE}_{s_{in}}$	$\text{RMSRE}_{s_{out}}$	$\text{RMSRE}_{k_{in}}$	$\text{RMSRE}_{k_{out}}$	RMSRE_p	RMSRE_w
K	10^{-3}	10^{-3}	0.40	0.40	0.0043	0.15
S	10^{-3}	10^{-3}	0.40	0.18	0.0054	0.15
D	10^{-3}	10^{-3}	0.39	0.39	0.0028	0.15
B	10^{-2}	10^{-2}	0.16	0.18	0.0015	0.15
C	10^{-2}	10^{-2}	0.39	0.36	0.0018	0.13
R	10^{-3}	10^{-3}	7.3	7.4	0.98	0.14

Model	Recall	β_{in}	β_{out}	$\text{MSE}_{l(d)}$	M
K	0.36	1.39(5)	1.39(7)	47.1	0.0
S	0.30	1.78(7)	1.78(8)	84.5	0.0
D	0.41	1.37(4)	1.37(5)	43.3	0.0
B	0.73	1.0(8)	1.0(8)	3.58	0.63
C	0.61	1.0(0)	1.0(0)	2.60	0.63
R	0.019	(0)	(4)	194	0.0

Supplementary Table 4. Reconstruction of network properties for the airport dataset. Values of Root Mean Squared Relative Error $\sqrt{n^{-1}\sum_i(\langle y_i\rangle/y_i - 1)^2}$ of reconstructed quantities $\langle y_i\rangle$ against empirical observations y_i, where y values are averages over histogram bins. RMSRE_s of model versus empirical in/out-strengths; RMSRE_k of model versus empirical in/out-degrees; RMSRE_p of empirical link probability versus model link probability; RMSRE_w of model versus empirical weights. Recall values for link prediction, obtained using the model link probability to determine the outcome of a binary classification test on the existence of a link. OLS fit exponent β of the strengths vs degrees relation; Mean Squared Error (MSE) between empirical and model curve of link probability as a function of distance; average modularity M of the network. For real data: $\beta_{in} = 0.9(1)$, $\beta_{out} = 0.9(2)$, $M = 0.64$

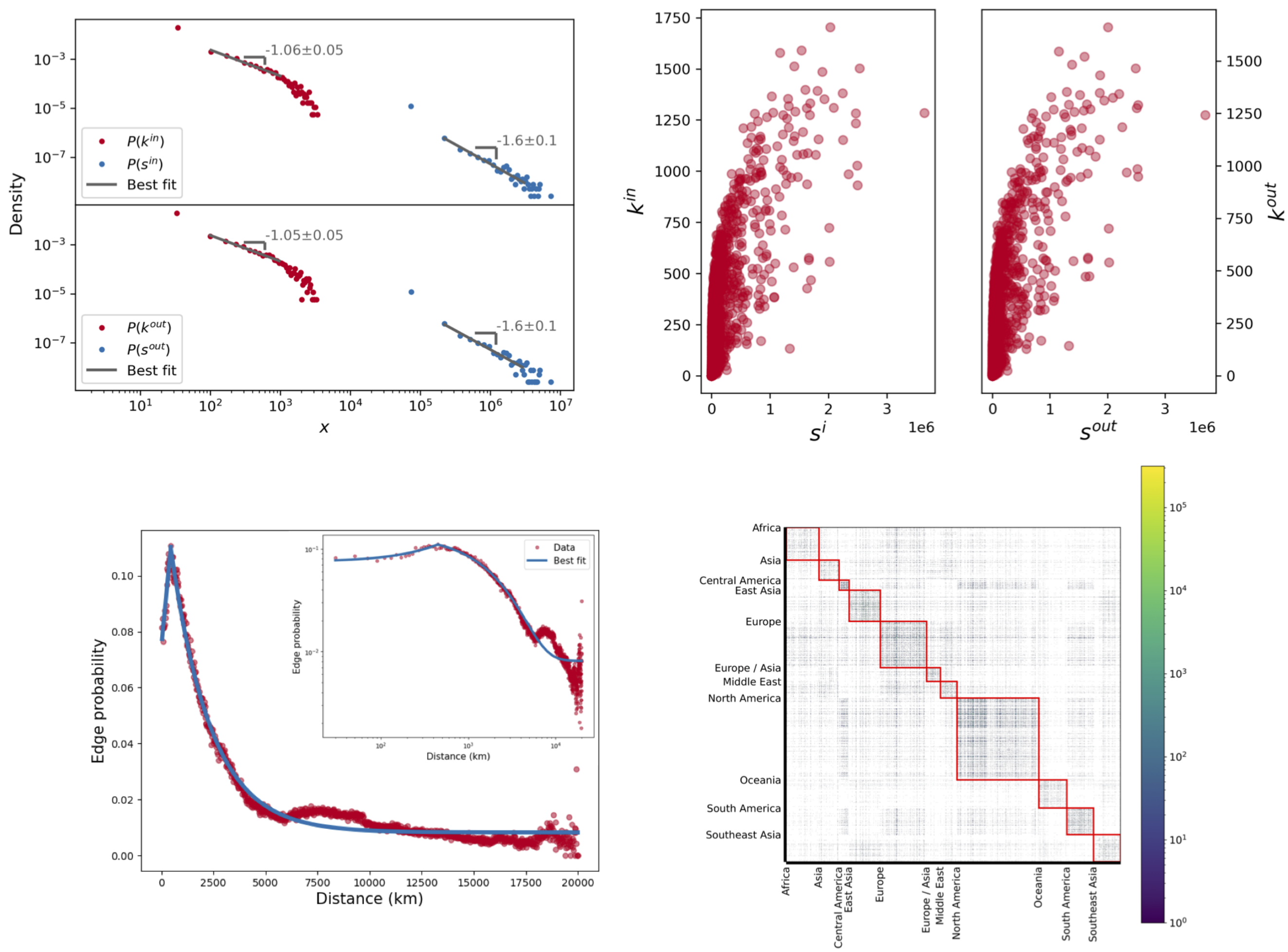

Supplementary Figure 7. Topological features of the WAN (airport dataset). (A) In/Out-Degree and In/Out-Strength distributions. (B) In/Out-Degrees as a function of In/Out-Strengths, with OLS fit exponents $\beta_{in} = 0.9(1)$ and $\beta_{out} = 0.9(2)$. (C) Link probability as a function of distance. Best fit parameters for the linear part: $d^* = 450km$, $m = 5.(9) \cdot 10^{-5}$ and $q = 7.(9) \cdot 10^{-2}$ ($R^2 = 0.83$); for the exponential decay: $d_0 = -38.(8) \cdot 10^2$, $a = 52.(5) \cdot 10^{-5}$, $b = 8.(2) \cdot 10^{-3}$ ($R^2 = 0.98$). (D) Block structure of the network, with modularity $M = 0.64$.

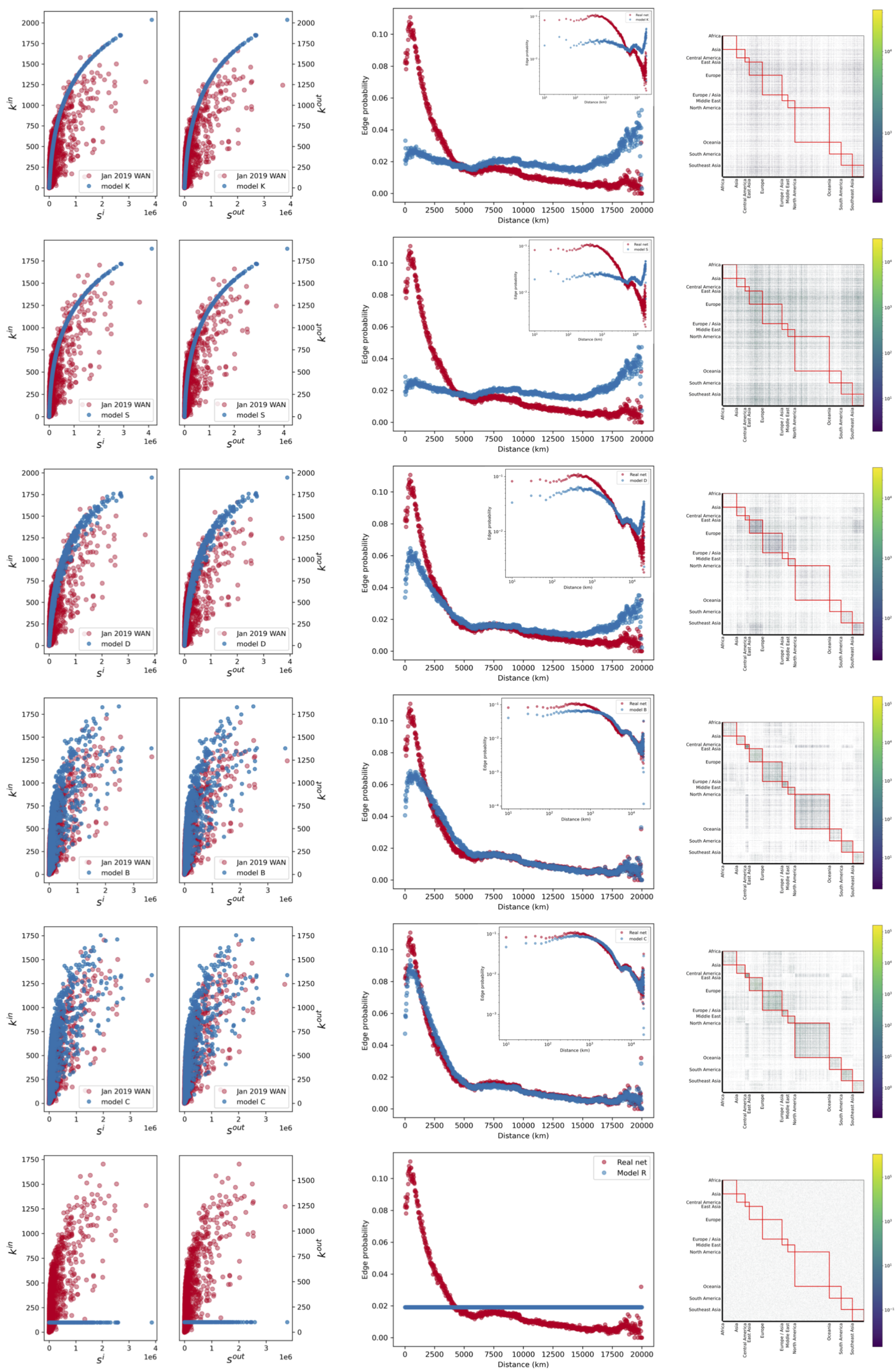

Supplementary Figure 8. Properties of the model networks for the airport dataset: degree vs strength, edge probability as a function of distance, community structure.

Supplementary Note 3

Results for ICAO regional communities

While we do not optimize models **B** and **C** with respect to the network partition into geographic regions, accuracy does depend on the imposed community structure. Here we show reconstruction results for a coarser partition based on ICAO's regional division (https://www.icao.int/present-regional-structure).

The standard ICAO classification comprises a total of 9 different regions: Africa, Asia, Caribbean, Europe, Middle East, North America, North Atlantic, Pacific and South America. Since this division results in some regions (e.g., Pacific) having too few airports, we merge these regions into the nearest denser ones. This produces a partition into 6 geographical regions: Africa, Asia/Pacific, Europe, North America, Middle East, Latin America & Caribbean (Supplementary Figure 9).

This partition is coarser than the one used in the main paper and induces a smaller value of modularity ($M = 0.58$). Thus by definition it decreases the performance of the model, which now uses less information.

Supplementary Table 5 report results for models **B, C** that exploit the network community structure. Comparing with Supplementary Table 2 we see that both models retain higher performance than models without communities (**K, S, D**) but their overall accuracy slighlty drops with respect to their implementation with the finer community structure used in the main paper. In particular, recall for model **B** drops by 33%, while model **C** is less affected as it exploits geographic distance as additional information.

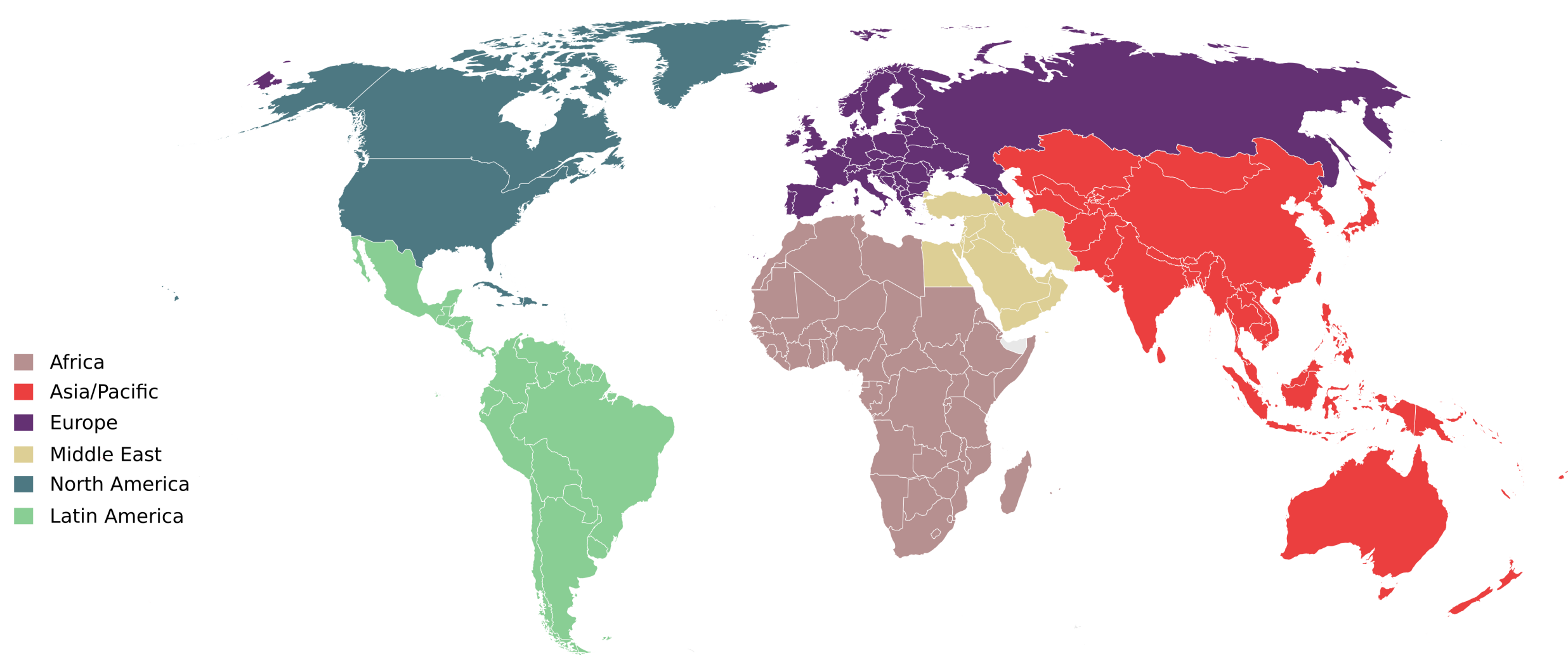

Supplementary Figure 9. World partition into ICAO-aggregated regions, inducing a network modularity of $M = 0.58$.

Models	RMSRE_k	RMSRE_p	RMSRE_w	Recall
B	0.21 (+0.03)	0.009 (-0.07)	0.136 (+0.073)	0.51 (-0.24)
C	0.31 (+0.05)	0.033 (+0.011)	0.156 (+0.067)	0.60 (-0.04)

Supplementary Table 5. Reconstruction of network properties for models informed on ICAO partition (differences in parenthesis are computed with respect to models informed on the finer partition of the main paper). Quantities are as defined in Supplementary Table 2.

Supplementary Note 4

Construction of the open dataset

Here we illustrate the procedure to build the open dataset and inform the model formulations accordingly. Firstly, we select all airports that appear in both OpenSky and Wikidata and map them to basins using geographic proximity, obtaining a reduced set of 836 basins (Supplementary Figure 10).

Topological information. From OpenSky we build an undirected binary network where $\tilde{a}_{ij} = 1$ denotes the presence of (at least one) direct flight between basins i and j in the period Sept.-Nov. 2019. This information is used to compute the total number of network links $\tilde{L}_{tot} = \sum_{i,j} \tilde{a}_{ij}$, required for models **K, S, D**, and the number of links among communities $\tilde{L}_{g,g'} = \sum_{i\in g}\sum_{j\in g'} \tilde{a}_{ij}$, required for models **B, C**. For model **D** we also compute $\tilde{l}(d)$, the probability of having a link between nodes at distance d, which agrees with the one computed on the OAG dataset (Supplementary Figure 11A). For model **S** we use as optimal exponent $\alpha = 0.8$ (the one computed for OAG data). Because connectivity obtained from OpenSky direct flights is systematically underestimated with respect to OAG origin-destination data (Supplementary Figure 11B), we rescaled all link count values by the same factor $\eta = L_{tot}/\tilde{L}_{tot} \simeq 1.627$ before feeding them to models (L_{tot} is the number of links in the OAG dataset computed for the reduced basin sets). Note that this rescaling is not a necessary step but ensures consistency between OpenSky and OAG data and improves models performance.

Traffic statistics. From Wikidata we compute passenger traffic volume (PAX) $\tilde{s}_i$ for each basin i by summing values of all corresponding airports for the period Sept.-Nov. 2019 (Supplementary Figure 11C). In order to improve consistency with OD values from OAG, we filter out a handful of small outliers, resulting in the set of 793 basins we use in all the following analyses. PAX strengths $\{\tilde{s}_i\}$ are enough to inform models **K, S, D**, while for **B, C** we need passenger fluxes computed with respect to each community g, $\{\tilde{s}_{ig}\}$, which also allow computing the required normalizing factors $\tilde{W}_{gg'} = \sum_{i\in g} \tilde{s}_{ig'}$. We obtain them by splitting Wikidata strengths according to rescaled connectivity-by-region obtained from OpenSky. After computing, for each basin i, total OpenSky degree $\tilde{k}_i = \sum_j \tilde{a}_{ij}$ and degree with respect to each region g, $\tilde{k}_{ig} = \sum_{j\in g} \tilde{a}_{ij}$, we check that the superlinear relation $s \sim k^{\beta}$ (with $\beta = 1.22$ the value computed on OAG data) holds also for region-specific values (Supplementary Figure 11D). Then we obtain

$$\tilde{s}_{ig} = \zeta\, \tilde{s}_i \left(\frac{\tilde{k}_{ig}}{\tilde{k}_i}\right)^{\beta} \tag{7}$$

where ζ is a normalization factor to ensure that regional values sum up to the correct total: $\sum_g \tilde{s}_{ig} \equiv \tilde{s}_i$. These values closely reproduce their OAG counterparts (Supplementary Figure 11E).

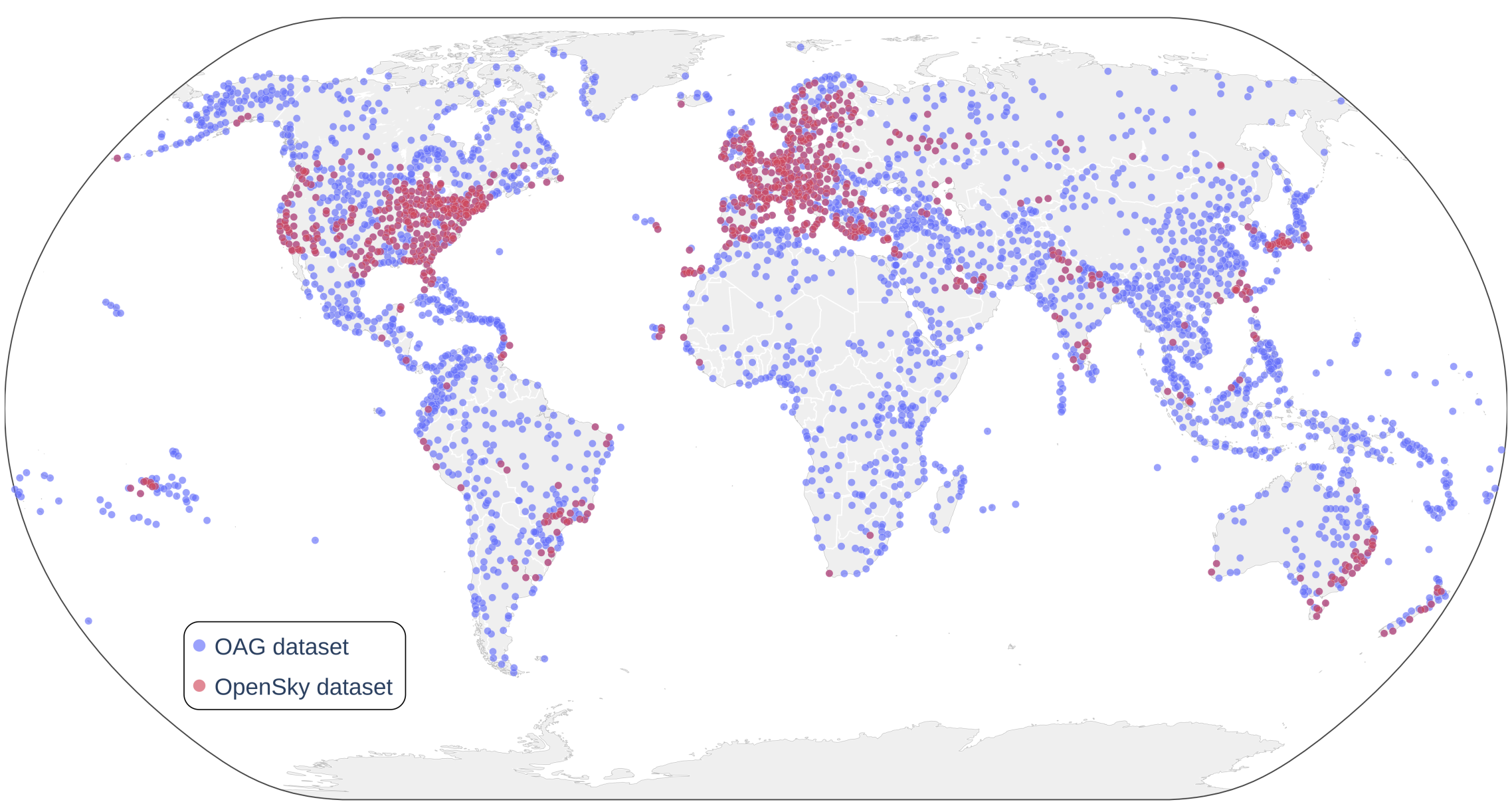

Supplementary Figure 10. Map of basins appearing in the open dataset (red points), with respect to all basins of the original OAG dataset (blue points). Basin coverage of the open data is not geographically uniform. In particular, only a few Chinese airports are present in the OpenSky data.

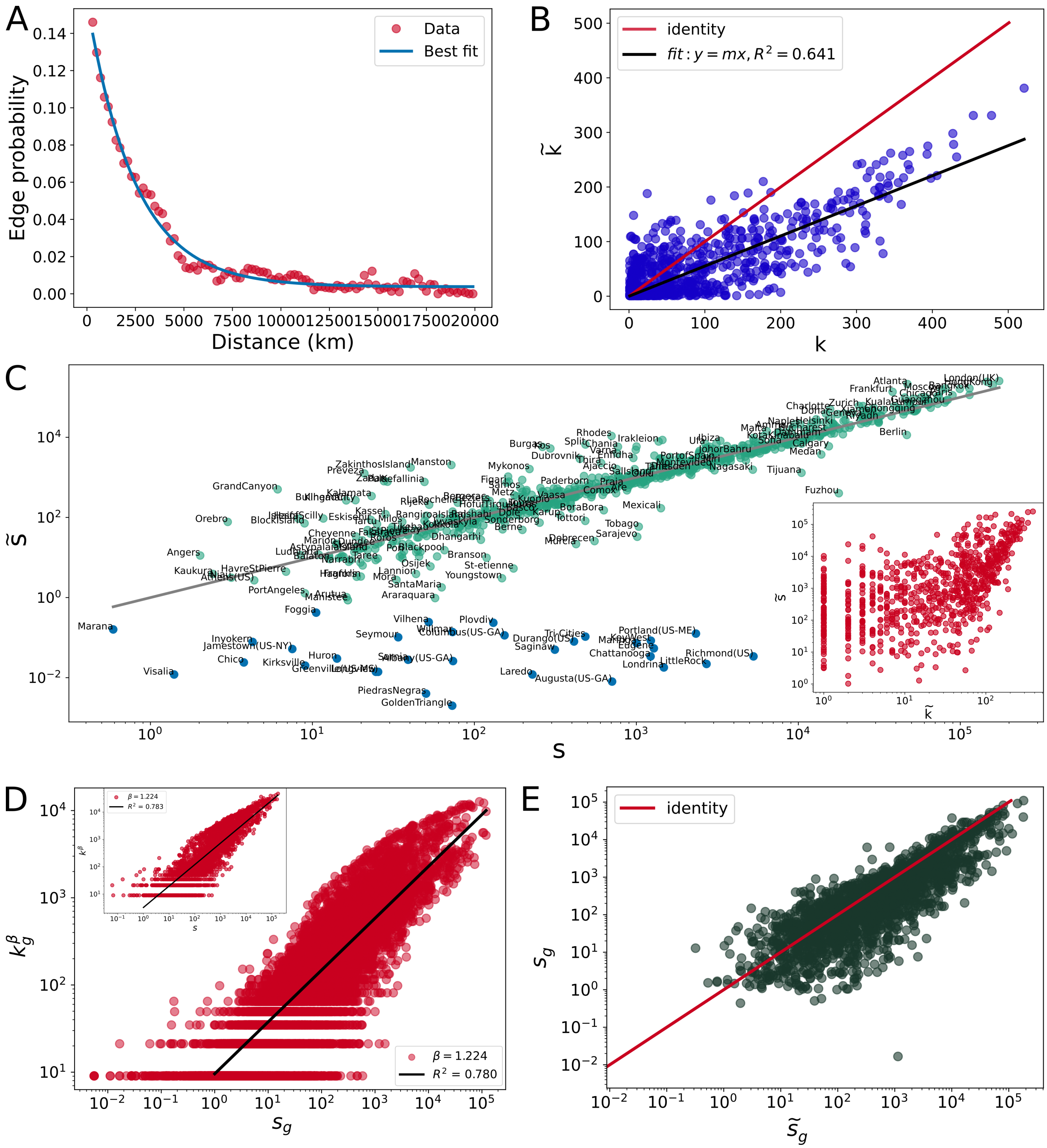

Supplementary Figure 11. **Features of the open dataset**. (A) Link probability as a function of geographic distance. The curve is fitted with an exponential function $\tilde{l}(d) = \exp[-a(d-d_0)] + b$. Best fit parameters are $d_0 = -4.(6) \cdot 10^3$, $a = 4.(1) \cdot 10^{-4}$, $b = -3.(8) \cdot 10^{-3}$ ($R^2 = 0.98$), hence the decay is quantitatively similar to that observed for OAG data (Figure 1C of the main text). (B) Relation between degrees computed on OpenSky ($\tilde{k}$) and those computed on the filtered OAG dataset (k). The best linear fit has slope $m = 0.5(5)$. (C) Relation between strengths computed on Wikidata ($\tilde{s}$) and those computed on the filtered OAG dataset (s). Outliers that are filtered out are colored in blue. Inset: fitness ansatz $\tilde{s}$ vs $\tilde{k}$ for the open dataset. (D) Relation between rescaled region-specific degrees k_g^β and strengths s_g^β for the filtered OAG data. Inset: Relation between total values k^β and s^β (same of Figure 1B of the main text). (E) Scatter plot of inferred region-specific strengths $\tilde{s}_g$ by eq. (7) and values computed on filtered OAG data s_g.

Results for models informed with open data

Here we discuss the results of all model formulations in reconstructing the topological and dynamical features of the WAN, when they are informed with the open dataset described above. As empirical benchmark, we consider the OAG data restricted to the selected set of 793 basins that are present in the open dataset. Supplementary Tables 6 and 7 contain an overview of the results, to be compared with Supplementary Tables 2 and 3 for models informed on the full OAG basin dataset.

Concerning topological properties (Supplementary Table 6), by construction all models reproduce the strength sequence $\tilde{s}$ as measured by PAX numbers, which is however different from OD counts s from the filtered OAG data (Supplementary Figure 11C), whence the non-zero values of RMSRE_s. In general, the reconstruction of degrees, connection probabilities and weights deteriorates when models are informed by open data, while recall of individual links increases (but for models **B** and **C**, see below). This apparent contradiction is explained by the largest basins having higher PAX $\tilde{s}$ with respect to OD counts because of many transit passengers. As such, connections between hubs get very high probability and, since they likely exist, they are also correctly recovered. In all other cases, however, models make more mistakes, resulting in a generally worse degree reconstruction.

Errors on link probabilities and weights for models **B** and **C** are slightly higher than for other models, due to a structural mismatch between OpenSky and OAG data at the regional level. When there are no links in the OpenSky data between node i and region g, by construction $\tilde{s}_{ig} = 0$ and the two become disconnected in the model ensembles. Then if a link from i to g does exist in the filtered OAG data, it cannot be recovered by construction (this situation cannot arise if models are directly informed on OAG data). Hence block models are able to reconstruct only 80.42% of all possible links of the filtered OAG dataset, with respect to a 100% link coverage obtained with non-block models. Despite this limitation, block models achieve the best performance with respect to aggregate quantities (strengths and degrees) as well as recall of individual links. Note that by considering only the accessible link set, recall increases to 0.73 and 0.78 for models **B** and **C**, respectively.

Another interesting aspect is that model **D** now becomes a close competitor of model **B**, and model **C** combining their features wins on **B** for some of the considered metrics. This suggests that distance plays a more important role in the network formation when connections are represented at the level of individual flight legs, rather than origin-destination paths.

In terms of epidemic simulations, comparison with results for the whole OAG dataset is not straightforward, because the filtered data comprises roughly 25% of all basins. Also, excluded nodes may have very large populations, especially for regions that are less covered like Asia. Seed airports are also slightly different (8 out of 24 changes): we have to replace Cairo, Algiers, Shanghai, Beijing, Santiago with Casablanca, Tunis, Barcelona, Brasilia, Buenos Aires. Yet, as Supplementary Table 7 shows, with open data the quality of simulations on synthetic networks decreases only slightly. Additionally, while the gap between the intrinsic stochastic variability of empirical data and the difference between model and empirical scenarios increases, values for models **B** and **C** remain statistically compatible with empirical ones.

Models	$RMSRE_s$	$RMSRE_k$	$RMSRE_p$	$RMSRE_w$	Recall	β	$MSE_{l(d)}$	M
K	11.6	0.44	0.047	2.11	0.47	1.3(6)	6.30	0.0
S	11.6	0.33	0.026	2.11	0.44	1.6(3)	9.08	0.0
D	11.6	0.41	0.011	2.11	0.54	1.3(6)	4.79	0.0
B	8.36	0.28	0.057	4.53	0.58	1.3(8)	0.56	0.18
C	8.36	0.35	0.070	4.53	0.62	1.1(6)	0.32	0.18
R	11.6	16	0.89	2.11	0.067	(0)	22.7	0.0

Supplementary Table 6. Reconstruction of network properties for model informed on the open dataset. For real data, $\beta = 1.2(4)$ and $M = 26$. Quantities are as defined in Supplementary Table 2.

Model	Δ area	L2[i]	Peak time	Peak magn.	Epid. length	RMSE[i_m]	L2[H]
K	2.5 ± 0.6	0.35 ± 0.08	8.5 ± 2.8	0.028 ± 0.012	15.2 ± 3.7	0.011 ± 0.001	17.8 ± 4.3
S	2.4 ± 0.6	0.34 ± 0.08	8.3 ± 2.8	0.025 ± 0.011	13.6 ± 4.1	0.011 ± 0.001	17.6 ± 3.9
D	2.3 ± 0.5	0.33 ± 0.07	8.0 ± 2.7	0.024 ± 0.011	11.9 ± 3.6	0.011 ± 0.001	16.7 ± 4.3
B	1.2 ± 0.5	0.17 ± 0.07	4.3 ± 2.4	0.0071 ± 0.0049	7.2 ± 2.4	0.0086 ± 0.0010	13.2 ± 2.8
C	1.3 ± 0.5	0.18 ± 0.07	4.4 ± 2.4	0.0079 ± 0.0059	10.5 ± 3.6	0.0085 ± 0.0010	12.6 ± 2.9
Data	0.55 ± 0.34	0.076 ± 0.047	1.7 ± 1.4	0.0042 ± 0.0039	3.3 ± 2.7	0.0055 ± 0.0010	7.4 ± 3.6

Supplementary Table 7. Similarity of simulations on real and model networks for the open dataset. Quantities are as defined in Supplementary Table 3. Model **R** is not shown as it often generates configurations where seed basins are disconnected, preventing disease propagation and thus leading to completely different epidemic outcomes).

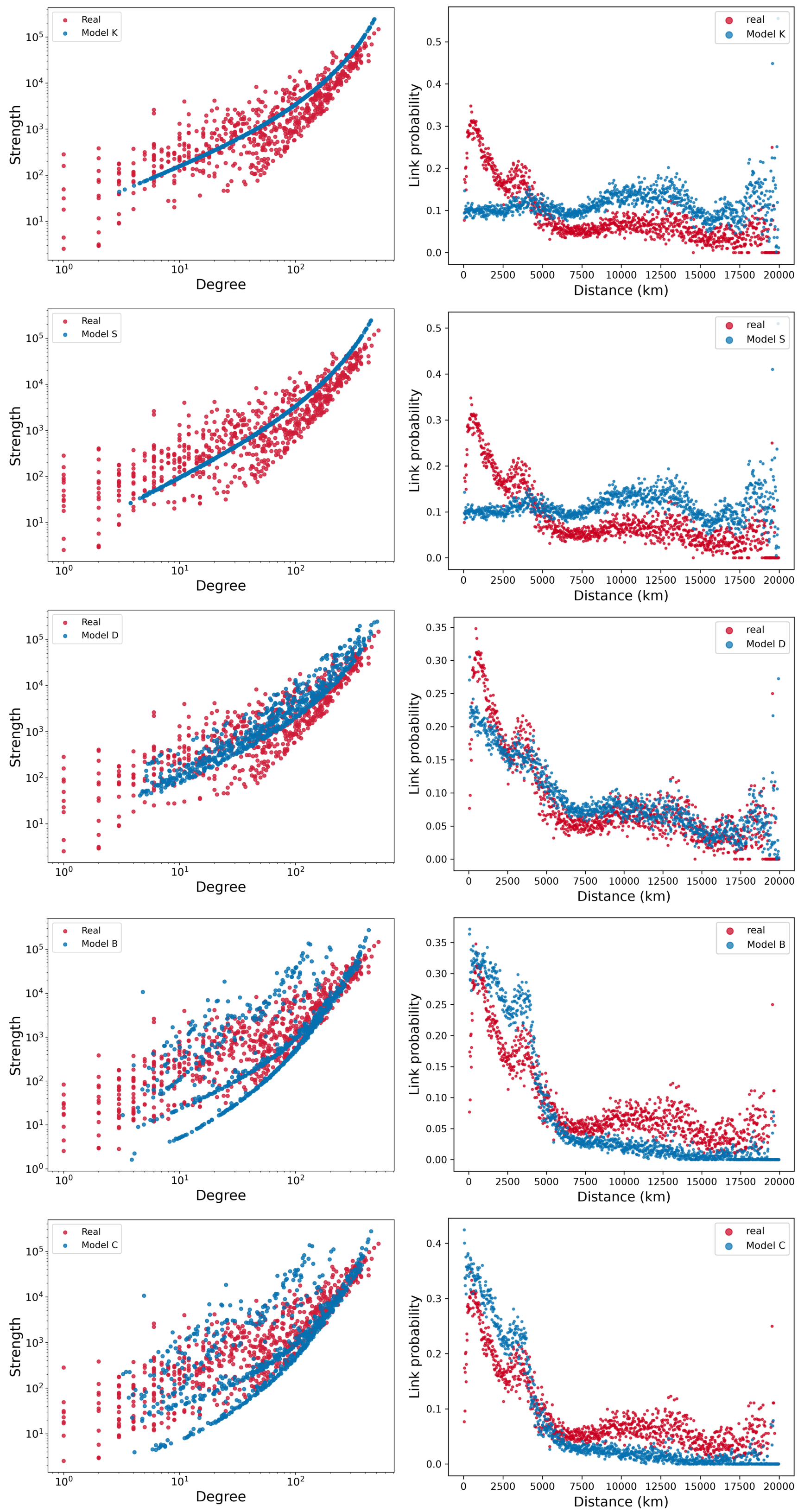

Supplementary Figure 12. Reconstruction of network properties of the filtered OAG network with open-data informed models. From top to bottom: Models **K**, **S**, **D**, **B**, **C**. Left column: Degrees versus Strengths relation; Right column: Link probability as a function of distance.

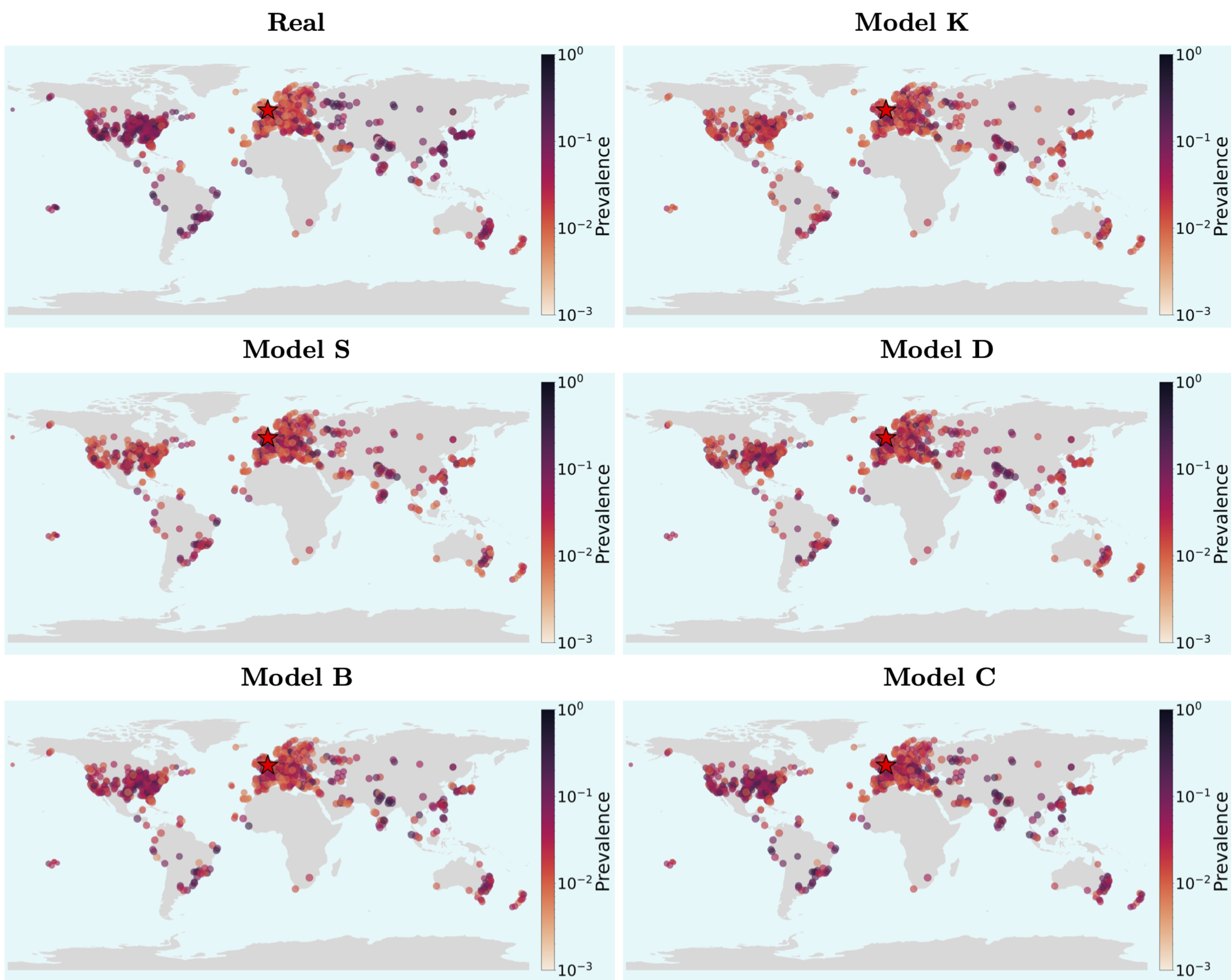

Supplementary Figure 13. Prevalence maps for the filtered OAG network: empirical data and open-data informed model reconstruction. Simulations with seed=London, at time step $t = 80$.

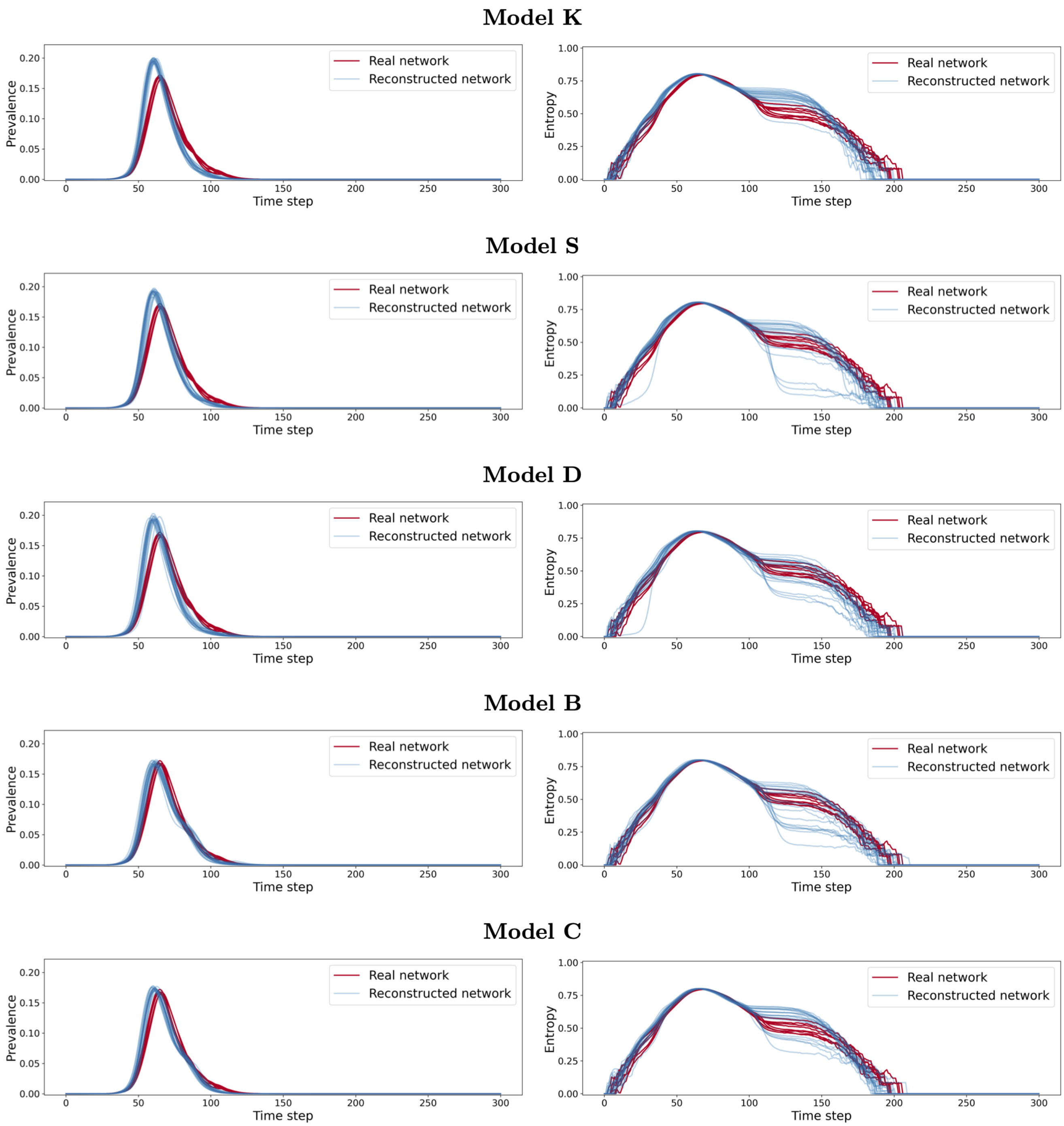

Supplementary Figure 14. Metapopulation results for the filtered OAG network: empirical data and open-data informed model reconstruction. Comparison of one real simulation and 20 model simulations, in terms of the time evolution of total prevalence and entropy of the prevalence distribution. Seed = London.

References

1. Official aviation guide (oag) worldwide flight and passenger data (2019).
2. Balcan, D. *et al.* Multiscale mobility networks and the spatial spreading of infectious diseases. *Proc. Natl. Acad. Sci.* **106**, 21484–21489, DOI: 10.1073/pnas.0906910106 (2009).
3. Cimini, G., Squartini, T., Garlaschelli, D. & Gabrielli, A. Systemic risk analysis on reconstructed economic and financial networks. *Sci. Reports* **5**, 15758, DOI: 10.1038/srep15758 (2015).
4. Mistrulli, P. E. Assessing financial contagion in the interbank market: Maximum entropy versus observed interbank lending patterns. *J. Bank. & Finance* **35**, 1114–1127, DOI: 10.1016/j.jbankfin.2010.09.018 (2011).
5. Squartini, T., Caldarelli, G., Cimini, G., Gabrielli, A. & Garlaschelli, D. Reconstruction methods for networks: The case of economic and financial systems. *Phys. Reports* **757**, 1–47, DOI: 10.1016/j.physrep.2018.06.008 (2018).
6. Barrat, A., Barthélemy, M., Pastor-Satorras, R. & Vespignani, A. The architecture of complex weighted networks. *Proc. Natl. Acad. Sci.* **101**, 3747–3752, DOI: 10.1073/pnas.0400087101 (2004).
7. Guimerà, R. & Amaral, L. A. N. Modeling the world-wide airport network. *The Eur. Phys. J. B - Condens. Matter* **38**, 381–385, DOI: 10.1140/epjb/e2004-00131-0 (2004).
8. Bagler, G. Analysis of the airport network of india as a complex weighted network. *Phys. A: Stat. Mech. its Appl.* **387**, 2972–2980, DOI: 10.1016/j.physa.2008.01.077 (2008).
9. Bianconi, G. The entropy of randomized network ensembles. *Europhys. Lett.* **81**, 28005, DOI: 10.1209/0295-5075/81/28005 (2008).
10. Bianconi, G., Pin, P. & Marsili, M. Assessing the relevance of node features for network structure. *Proc. Natl. Acad. Sci.* **106**, 11433–11438, DOI: 10.1073/pnas.0811511106 (2009).
11. Bianconi, G. *Information theory of spatial network ensembles*, chap. 5 (Edward Elgar Publishing, 2021).
12. Zanin, M. & Lillo, F. Modelling the air transport with complex networks: A short review. *The Eur. Phys. J. Special Top.* **215**, 5–21, DOI: 10.1140/epjst/e2013-01711-9 (2013).
