# Supplementary material for: Block-fitness modeling of the global air mobility network
Source: Nat Commun. 2026 Oct 12;17:11559. doi: 10.1038/s41467-026-78115-6 (PMC13748577; doi:10.1038/s41467-026-78115-6)
Supplement: Supplementary file 2 [file 41467_2026_78115_MOESM2_ESM.pdf]

nature portfolio

# Peer Review File

# Block-Fitness Modeling of the Global Air Mobility Network

Corresponding Author: Professor Guido Caldarelli

**This file contains all reviewer reports in order by version, followed by all author rebuttals in order by version.**

Version 0:

Reviewer comments:

Reviewer #1

(Remarks to the Author)
In this manuscript, authors propose a generative model able to synthesise networks similar to the world air transportation one, which can then be used as input for, e.g., modeling epidemic spreading. The authors motivate this by the fact that open access data on this type of mobility is restricted, and is usually behind paywalls. This is partly true (see below), and I see the added value for the community - to be clear, I would personally use this model, if some problems were solved. Yet, I also think that the added value of the paper has to be improved, and several issues have to be tackled. I am listing below two major problems, and a set of minor comments.

Major problem 1: What, exactly?

Authors state that they "introduce a generative model of the WAN"; and the latter is defined as "a complex network of airports (nodes) connected by direct flights (links)". So, as a reader, I get the clear impression that authors are referring to the airport network. Yet, the generative model is then based on "the Official Aviation Guide (OAG), which reports monthly counts of origin-destination passengers between airports worldwide". These are two different things: airports connected by direct flights, vs. airports connected by passengers. That is, a passengers flying London -> New York -> Los Angeles would be counted as one link London -> Los Angeles in the latter; but the former would include two links, corresponding to the direct flights London -> New York and New York -> Los Angeles. I expect that the network that is really relevant from the point of view of epidemic spreading is the latter one; but authors state that they have a method to reconstruct the former...

I suspect that this is only a confusion in the terminology. I would personally not call the recovered networks (of pax travels) a WAN, especially when defining the WAN in the third paragraph as the network of directed flights. Or maybe, clarify that two networks can be called WAN, and explain which one the authors are referring to.

Major problem 2: What is this for?

Reading the abstract and introduction, the problem is quite well stated: several research problems use as input the mobility of passengers in air transport, but this information is usually behind paywalls (and quite expensive ones!). With this I absolutely resonate with the authors; and I've been working on additional problems, beyond those listed, in which these data would be very useful. So, as a solution, the authors propose this generative model. Up to here, everything is fine; actually, I am keen to test it.

The problem is that the generative model uses real data as input (which is fair); but these data are the ones from OAG, that the authors cannot redistribute, and that have to be bought. Then, my question is: if, in order to use the model, the user needs to have access to these data... what is the point of synthesising new ones? That is, if I have the real data, why should I use the model? And, if I don't have them, how can I use the model?

An answer to this last question is possibly provided by the authors at the end of the manuscript: "Our model is able to reconstruct the network from aggregate information without training on real instances, i.e, without ever seeing the full network." This, as far as I understand, refers to the strength of nodes, i.e. the number of passengers traveling to and from each airport. If this is so, would it be possible to run the model only using open-source data? As, for instance, pax numbers can easily be obtained from Wikipedia. Yet, this would require some additional discussions in the paper; for instance,

compare the results obtained with open data with the ones from OAG (as probably the latter are more precise); and even provide the corresponding data and code in the repository.

Yet, as it stands right now, the paper is really not useful. The authors present a generative model that cannot be used unless the paid data are available - and if these were available, then there is no need for the model. Which also means that results are not reproducible, unless one has the original data.

The main problem that I have with this contribution is again what I said above: "I am keen to test it". Yet, I cannot in its actual form, see also below the problems with the code. If I could download the model and create synthetic data, then I'd see the added value, and it would actually be an important one.

Minor problem: the provided code.

While I absolutely appreciate that the authors have provided a link to a repository with Python code, I have to say that its quality is not great. There is no documentation whatsoever. For instance, the input data are accessed through two files:
input_path = '/mnt/TANK4TB/Network_reconstruction/Airport_reconstruction/Data_codes_final/DIR/Input/'
output_path = '/mnt/TANK4TB/Network_reconstruction/Airport_reconstruction/Data_codes_final/DIR/Output/'
But there is no information about the format required.
In some cases, functions are documented with only one line of text, saying what they do - but not the expected inputs, the outputs, etc. And in most cases, that text is only a cryptic "# TO FINISH"...
To be clear, I am not expecting a professional code here, with Unit Testing and full documentation; but something more is required, otherwise the code is effectively useless.

Minor problem: origin-destinations and connections.

Something that I was expecting, from my (limited) knowledge of disease propagation models, is a high importance of connecting passengers. Back to the previous example, a passenger with COVID flying London -> New York -> Los Angeles will not only propagate the disease from London to LA, but also (potentially) to passengers that are taking flights in NY. I understand that this information is lost in the generated network? What is the impact of this? And, is there any way of recovering such intermediate connections?

Minor problem: The division into world regions.

I found it surprising that the authors have used this division, instead of the ICAO's regional division. I would have expected the latter to be more real, as it is designed with aviation in mind. But I also realised that it may introduce new problems - as, for instance, Europe is divided into two regions, "L" and "E". Still, it would be nice if authors could discuss the sensitivity of the proposed method to the used partition.

Reviewer #2

(Remarks to the Author)
This paper describes a data-based model for the pattern of passenger flows in the world air transportation network. Starting from monthly data on individual passenger trips, the authors first aggregate the data geographically based on origins and destinations, then use the results to fit a model of flows that preserves traffic volume in each region. They are able to predict with impressive accuracy the connections in the network ("link prediction"), and their model gives a good fit to a number of statistical distributions within the data. They give an example application to modeling the spread of an epidemic, comparing results on both the real air network and their simulated network, and agreement is excellent.

I think this is an impressive piece of work, and potentially valuable for future applications such as epidemiological modeling. It is potentially a promising candidate for publication but I do have some issues with the paper and its presentation. My main issue is that I feel the paper fails to make clear what the goals of the exercise are. The authors state that they want an "accurate representation of the world air transportation network". And yet their starting point is the OAG, which gives detailed counts of passenger numbers between origins and destinations, and hence is *already* a very complete description of the air transportation network. I assume that the authors' goal must to be add something to the data that is not already present, but they don't clearly state what this is. Reading between the lines, I infer that the general goal is to improve the time resolution of the data, extrapolating from the coarser monthly time-scale of the OAG to shorter time-scales, but the paper needs more clarity on this. Perhaps the authors' goal is to infer passenger flows between individual airport pairs, rather than complete origin-destination trips. Or they may want to develop an aggregate picture of geographic flows, in terms of the Voronoi tessellation they use. Perhaps they want to simulate passenger movement at the individual level, in the manner of an agent-based model. The closest the paper comes to spelling out its goals is when it speculates about a "generative framework capable of reproducing the structural and functional properties of the WAN from limited aggregate information". This hints potentially at agent-based approaches or high temporal resolution estimates.

I have a small technical question about the nature of the predicted flows, which was unclear to me from the paper. It appears that the model predicts number of passengers passing between regions or airports. In general, when (for example)

performing epidemiological simulations, one does not usually want direct passenger flows between individual regions or airports. Rather, one wants complete passenger itineraries from start to finish. If a passenger with the flu boards a plane in Paris and flies to LA via New York, then it is important that it is the *same* passenger. Indeed their touching down in New York may be only a minor detail, far less important than their final destination of LA in terms of disease spread. It was unclear to me from the paper whether the authors' model is able to predict accurately complete itineraries in this manner.

Overall, the technical achievements of the paper appear impressive. I believe that if the goals were more clearly articulated then this could be useful work that could lead to improved predictions in epidemic modeling and other areas. I would be happy to consider the paper again after revision.

Version 1:

Reviewer comments:

Reviewer #1

(Remarks to the Author)
I am extremely glad to see that authors have directly addressed, and in a very convincing way, all my comments.
The text is now clear, regarding the scope of the model (flights vs. passengers).
Most importantly, I tested the code and the model, and it works perfectly. The documentation the authors provide is enough to enable the use of other data sources - at least, if one knows a minimum about aeronautical data. I think this is a key point: now I clearly see the "business proposition", and personally I see several scenarios in which I will use the model.
Given this, I think the manuscript can be published now.

(Remarks on code availability)

Reviewer #2

(Remarks to the Author)
The authors have dealt thoroughly with the issues I raised in my previous review. In particular the paper now states clearly, in the abstract, in the introduction, and throughout the text, what the goals are, the nature of the model outputs, and the data on which its operation is based. I believe the paper is now suitable for publication and recommend its acceptance.

(Remarks on code availability)

**Open Access** This Peer Review File is licensed under a Creative Commons Attribution 4.0 International License, which permits use, sharing, adaptation, distribution and reproduction in any medium or format, as long as you give appropriate credit to the original author(s) and the source, provide a link to the Creative Commons license, and indicate if changes were made.

In cases where reviewers are anonymous, credit should be given to 'Anonymous Referee' and the source.
The images or other third party material in this Peer Review File are included in the article's Creative Commons license, unless indicated otherwise in a credit line to the material. If material is not included in the article's Creative Commons license and your intended use is not permitted by statutory regulation or exceeds the permitted use, you will need to obtain permission directly from the copyright holder.
To view a copy of this license, visit https://creativecommons.org/licenses/by/4.0/

Dear Editor,

We thank you and the reviewers for the careful assessment of our manuscript and for the constructive comments. The raised suggestions have helped us clarify the scope and contribution of the work, strengthen its practical relevance, and improve the manuscript and accompanying code.

All changes in the revised manuscript are highlighted in red. Our point-by-point responses are provided below.

Best regards,
The Authors

## Reviewer #1 (Remarks to the Author):

In this manuscript, authors propose a generative model able to synthesise networks similar to the world air transportation one, which can then be used as input for, e.g., modeling epidemic spreading. The authors motivate this by the fact that open access data on this type of mobility is restricted, and is usually behind paywalls. This is partly true (see below), and I see the added value for the community - to be clear, I would personally use this model, if some problems were solved. Yet, I also think that the added value of the paper has to be improved, and several issues have to be tackled. I am listing below two major problems, and a set of minor comments.

Major problem 1: What, exactly?

Authors state that they "introduce a generative model of the WAN"; and the latter is defined as "a complex network of airports (nodes) connected by direct flights (links)". So, as a reader, I get the clear impression that authors are referring to the airport network. Yet, the generative model is then based on "the Official Aviation Guide (OAG), which reports monthly counts of origin-destination passengers between airports worldwide". These are two different things: airports connected by direct flights, vs. airports connected by passengers. That is, a passengers flying London -> New York -> Los Angeles would be counted as one link London -> Los Angeles in the latter; but the former would include two links, corresponding to the direct flights London -> New York and New York -> Los Angeles. I expect that the network that is really relevant from the point of view of epidemic spreading is the latter one; but authors state that they have a method to reconstruct the former…

I suspect that this is only a confusion in the terminology. I would personally not call the recovered networks (of pax travels) a WAN, especially when defining the WAN in the third paragraph as the network of directed flights. Or maybe, clarify that two networks can be called WAN, and explain which one the authors are referring to.

Reply: We thank the reviewer for identifying this ambiguity. Our study concerns air transportation at the level of complete origin–destination (OD) passenger trips. Thus, a passenger travelling from London to Los Angeles via New York contributes to the OD flow from London to Los Angeles, rather than to a direct connection between London and New York and between New York and Los Angeles.

We have substantially revised the Introduction to distinguish clearly between the direct-flight network, whose links represent flight legs between airports, and the OD passenger network, whose links represent complete passenger journeys between an origin and a final destination. To avoid further ambiguity, we now define the object reconstructed by our model as the worldwide OD air-passenger network at the beginning of the Results section and use this terminology consistently throughout the manuscript.

We have also clarified that, for the metapopulation dynamics considered in this work, the relevant quantity is the number of individuals moving from a source population to their final destination. We discuss this point in the revised Methods section.

Major problem 2: What is this for?

Reading the abstract and introduction, the problem is quite well stated: several research problems use as input the mobility of passengers in air transport, but this information is usually behind paywalls (and quite expensive ones!). With this I absolutely resonate with the authors; and I've been working on additional problems, beyond those listed, in which these data would be very useful. So, as a solution, the authors propose this generative model. Up to here, everything is fine; actually, I am keen to test it.

The problem is that the generative model uses real data as input (which is fair); but these data are the ones from OAG, that the authors cannot redistribute, and that have to be bought. Then, my question is: if, in order to use the model, the user needs to have access to these data... what is the point of synthesising new ones? That is, if I have the real data, why should I use the model? And, if I don't have them, how can I use the model?

An answer to this last question is possibly provided by the authors at the end of the manuscript: "Our model is able to reconstruct the network from aggregate information without training on real instances, i.e, without ever seeing the full network." This, as far as I understand, refers to the strength of nodes, i.e. the number of passengers traveling to and from each airport. If this is so, would it be possible to run the model only using open-source data? As, for instance, pax numbers can easily be obtained from Wikipedia. Yet, this would require some additional discussions in the paper; for instance, compare the results obtained with open data with the

ones from OAG (as probably the latter are more precise); and even provide the corresponding data and code in the repository.

Yet, as it stands right now, the paper is really not useful. The authors present a generative model that cannot be used unless the paid data are available - and if these were available, then there is no need for the model. Which also means that results are not reproducible, unless one has the original data.

The main problem that I have with this contribution is again what I said above: "I am keen to test it". Yet, I cannot in its actual form, see also below the problems with the code. If I could download the model and create synthetic data, then I'd see the added value, and it would actually be an important one.

Reply: We thank the reviewer for raising this central issue. We developed our model as an interpretable generative framework that serves two complementary purposes: it provides insight into the mechanisms shaping the OD air-passenger network, and it enables reconstruction of that network when detailed airport-pair OD data are unavailable but aggregate information can be obtained. We now make this point more explicit in the Introduction and Conclusion.
As the referee correctly noted, in the original implementation the model is informed with input aggregate quantities derived from the OAG data, which we cannot redistribute. Therefore, following the reviewer's suggestion, we additionally implemented and tested an end-to-end reconstruction pipeline based on openly available data. We constructed an open dataset by combining public flight-record data from OpenSky, used to extract network-topological information, with publicly available airport passenger-traffic statistics from Wikipedia, used to estimate airport-level traffic strengths. We then compared the reconstructed networks with the corresponding empirical OAG OD network for the airports represented in the open dataset. The results show that the model remains accurate when informed by imperfect but fully open aggregate information. We have added these results to the main text (before Conclusion), with details on data construction and additional analyses in Supplementary Sections S6 and S7. The corresponding open data and code are now included in the public repository.

We believe that this addition demonstrates the practical use case more clearly: researchers without access to proprietary airport-pair OD matrices can use openly available aggregate data to construct a realistic approximation of the OD air-passenger network for applications such as network-based epidemic simulations.

Minor problem: the provided code.

While I absolutely appreciate that the authors have provided a link to a repository with Python code, I have to say that its quality is not great. There is no documentation whatsoever. For instance, the input data are accessed through two files:
input_path =
'/mnt/TANK4TB/Network_reconstruction/Airport_reconstruction/Data_codes_final/DIR/Input/'

output_path =
'/mnt/TANK4TB/Network_reconstruction/Airport_reconstruction/Data_codes_final/DIR/Output/'
But there is no information about the format required.
In some cases, functions are documented with only one line of text, saying what they do - but not the expected inputs, the outputs, etc. And in most cases, that text is only a cryptic "# TO FINISH"...
To be clear, I am not expecting a professional code here, with Unit Testing and full documentation; but something more is required, otherwise the code is effectively useless.

Reply: We agree that the original repository did not provide sufficient documentation for third-party use. We have therefore reorganized the repository and added a README and user documentation describing the required inputs, data formats, workflow, outputs, and steps required to reproduce the analyses. The revised repository also includes the open data used in the new reconstruction exercise and the associated scripts.

Minor problem: origin-destinations and connections.

Something that I was expecting, from my (limited) knowledge of disease propagation models, is a high importance of connecting passengers. Back to the previous example, a passenger with COVID flying London -> New York -> Los Angeles will not only propagate the disease from London to LA, but also (potentially) to passengers that are taking flights in NY. I understand that this information is lost in the generated network? What is the impact of this? And, is there any way of recovering such intermediate connections?

Reply: We thank the reviewer for this useful question. Our model works at the level of OD passenger flows, meaning that a passenger travelling from Paris to Los Angeles via New York is represented as travelling from Paris to Los Angeles. This is the appropriate representation for the population-level epidemic simulations considered in our study, where the central quantity is the number of individuals relocating from a source population to their final destination.
Using traffic on individual flight legs instead would assign connecting travellers to transit hubs and can overestimate their contribution to importation risk at those hubs while underestimating the risk at their final destinations. OD matrices are therefore commonly used in metapopulation models of disease importation and spatial spread.
Transmission risk during transit is typically neglected with respect to risk at the destination for several reasons. First, empirical evidence consistently shows in-flight transmission of respiratory pathogens is rare, thanks to high cabin air exchange rates and HEPA filtration [Hertzber et al., PNAS 2018 (115(14):3623–3627]. Second, most infected travelers are in the latent stage when they board, so the epidemiologically relevant event is progression to infectiousness after arrival at the destination. Third, transit dwell times are orders of magnitude shorter than time spent at the destination, making the probability of seeding a sustained chain at an intermediate hub much less likely. We added the above explanation in the Methods section (epidemic simulations).

Minor problem: The division into world regions.

I found it surprising that the authors have used this division, instead of the ICAO's regional division. I would have expected the latter to be more real, as it is designed with aviation in mind. But I also realised that it may introduce new problems - as, for instance, Europe is divided into two regions, "L" and "E". Still, it would be nice if authors could discuss the sensitivity of the proposed method to the used partition.

<u>Reply</u>: We agree that the choice of geographic partition affects reconstruction performance. Our method is not optimized for a specific regional division: a coarser partition necessarily provides less information about interregional flows and can therefore reduce accuracy.

To assess this sensitivity, we added a new implementation based on the ICAO regional division in Supplementary Section S5. As expected, the coarser partition yields lower performance than the more detailed partition used in the main analysis; nevertheless, reconstruction accuracy remains high. We now discuss this dependence explicitly in the manuscript.

## Reviewer #2 (Remarks to the Author):

This paper describes a data-based model for the pattern of passenger flows in the world air transportation network. Starting from monthly data on individual passenger trips, the authors first aggregate the data geographically based on origins and destinations, then use the results to fit a model of flows that preserves traffic volume in each region. They are able to predict with impressive accuracy the connections in the network ("link prediction"), and their model gives a good fit to a number of statistical distributions within the data. They give an example application to modeling the spread of an epidemic, comparing results on both the real air network and their simulated network, and agreement is excellent.

I think this is an impressive piece of work, and potentially valuable for future applications such as epidemiological modeling. It is potentially a promising candidate for publication but I do have some issues with the paper and its presentation. My main issue is that I feel the paper fails to make clear what the goals of the exercise are. The authors state that they want an "accurate representation of the world air transportation network". And yet their starting point is the OAG, which gives detailed counts of passenger numbers between origins and destinations, and hence is *already* a very complete description of the air transportation network. I assume that the authors' goal must to be add something to the data that is not already present, but they don't clearly state what this is. Reading between the lines, I infer that the general goal is to improve the time resolution of the data, extrapolating from the coarser monthly time-scale of the OAG to shorter time-scales, but the paper needs more clarity on this. Perhaps the authors' goal is to

infer passenger flows between individual airport pairs, rather than complete origin-destination trips. Or they may want to develop an aggregate picture of geographic flows, in terms of the Voronoi tessellation they use. Perhaps they want to simulate passenger movement at the individual level, in the manner of an agent-based model. The closest the paper comes to spelling out its goals is when it speculates about a "generative framework capable of reproducing the structural and functional properties of the WAN from limited aggregate information". This hints potentially at agent-based approaches or high temporal resolution estimates.

Reply: We thank the reviewer for this comment and apologize that the original manuscript did not state the objective with sufficient precision. The aim of this work is to develop and validate an interpretable generative model that reconstructs the airport-pair OD passenger network from aggregate information that is in principle easier to access than detailed traffic data between airports. Indeed the model is conditioned on airport-level traffic totals and aggregate regional flows, generating detailed OD synthetic networks consistent with those constraints. The revised manuscript now makes this more explicit in the Introduction and Conclusion.

We remark that the model does not aim to infer individual passenger itineraries, increase the temporal resolution of the data, or simulate agent-level trajectories. Its output is a reconstructed monthly OD passenger-flow network. This network can subsequently be used as input for population-level applications, including epidemic-spreading models.

The OAG data provide a detailed OD matrix and are therefore used as a restricted-access benchmark for assessing the accuracy of the model, when informed with true aggregate information on the network.
To further demonstrate that the framework can be used when proprietary OD data are unavailable, we added a new analysis based entirely on openly available aggregate information. Public flight-record data are used to obtain network-topological information, while public airport passenger statistics are used to estimate airport-level traffic strengths. The resulting reconstructed networks are then evaluated against the empirical OAG network for the airports represented in the open dataset.

These results, now included in the main text and Supplementary Sections S6 and S7, show that the model remains accurate when informed with openly available but imperfect aggregate data. The associated data and code have been added to the public repository.

I have a small technical question about the nature of the predicted flows, which was unclear to me from the paper. It appears that the model predicts number of passengers passing between regions or airports. In general, when (for example) performing epidemiological simulations, one does not usually want direct passenger flows between individual regions or airports. Rather, one wants complete passenger itineraries from start to finish. If a passenger with the flu boards a plane in Paris and flies to LA via New York, then it is important that it is the *same* passenger. Indeed their touching down in New York may be only a minor detail, far less important than their

final destination of LA in terms of disease spread. It was unclear to me from the paper whether the authors' model is able to predict accurately complete itineraries in this manner.

Reply: Our model predicts complete OD passenger flows. Thus, a passenger travelling from Paris to Los Angeles via New York contributes to the OD connection between Paris and Los Angeles, rather than being treated as an independent passenger movement on each flight leg. This is the representation used in our epidemic simulations, because the relevant quantity for the population-level importation process is the number of individuals moving between their origin and final-destination populations. We have revised the manuscript to state this explicitly and to distinguish OD flows from direct-flight traffic throughout.
We thank the reviewer for noting that this point was unclear in the original presentation. We believe that the revised text now makes both the model's output and its epidemiological interpretation substantially clearer.

Overall, the technical achievements of the paper appear impressive. I believe that if the goals were more clearly articulated then this could be useful work that could lead to improved predictions in epidemic modeling and other areas. I would be happy to consider the paper again after revision.
